# Noninvasive respiratory support following extubation in critically ill adults with obesity: a systematic review and network meta-analysis

**DOI:** 10.1016/j.eclinm.2024.103002

**Published:** 2024-12-16

**Authors:** Joris Pensier, Arthur Naudet-Lasserre, Clément Monet, Mathieu Capdevila, Yassir Aarab, Inès Lakbar, Gérald Chanques, Nicolas Molinari, Audrey De Jong, Samir Jaber

**Affiliations:** a Department of Anesthesia and Intensive Care Unit, Regional University Hospital of Montpellier, St-Eloi Hospital, University of Montpellier, Montpellier, CEDEX 5, France; b PhyMedExp, University of Montpellier, INSERM U1046, CNRS UMR, 9214, Montpellier, France; c Medical Information, IMAG, CNRS, Univ Montpellier, Centre Hospitalier Regional Universitaire de Montpellier, Montpellier, France; d Institut Desbrest de Santé Publique (IDESP) INSERM - Université de Montpellier, Département d'informatique Médicale, CHRU Montpellier, France

**Keywords:** Noninvasive ventilation, BIPAP, CPAP, High-flow nasal cannula oxygen, HFNO, ICU, Obesity

## Abstract

**Background:**

Patients with obesity are at high-risk of extubation failure. Discrepancies were found in the results of recent randomized controlled trials (RCTs) regarding the roles of noninvasive ventilation (NIV), high flow nasal cannula (HFNC) and conventional oxygen therapy (COT) to prevent extubation failure in critically ill patients with obesity.

**Methods:**

In this systematic review and network meta-analysis, we searched MEDLINE, Cochrane Center Register of Controlled Trials and Web of Science from 1 January 1998 to 1 July 2024 for RCTs evaluating noninvasive respiratory support therapies (NIV, HFNC, COT, NIV + HFNC) after extubation in critically ill adults with obesity. Primary outcome was reintubation at day 7. Secondary outcome was 28-day mortality. We generated pooled risk ratios (RR) and numbers needed to treat (NNT). We rated risk of bias using the Cochrane risk-of-bias 2.0 tool. The study was registered with PROSPERO (CRD 42022308995).

**Findings:**

In seven RCTs including 1933 patients, NIV + HFNC (RR 0.36 [95% confidence interval (CI) 0.16–0.82], NNT = 10 [95% CI 7–33]) and NIV (RR 0.45 [95% CI 0.23–0.88], NNT = 11 [95% CI 8–50]) but not HFNC (RR 0.79 [95% CI 0.40–1.59]) reduced reintubation at day 7, compared to COT. Compared to HFNC, NIV + HFNC (RR 0.46 [95% CI 0.23–0.90], NNT = 14 [95% CI 10–77]) but not NIV (RR 0.57 [95% CI 0.32–1.02]) reduced reintubation at day 7. Compared to HFNC, both NIV (RR 0.31 [95% CI 0.13–0.74], NNT = 15 [95% CI 12–40]) and NIV + HFNC (RR 0.30 [95% CI 0.10–0.89], NNT = 15 [95% CI 11–90]) reduced 28-day mortality.

**Interpretation:**

The results suggest that compared to COT and HFNC, NIV alone or with HFNC reduces reintubation in critically ill patients with obesity after extubation. Compared to HFNC, NIV alone or with HFNC reduces mortality. The number needed to treat with NIV or NIV + HFNC to avoid one death was 15. These findings support the application of NIV to mitigate extubation failure in critically ill adults with obesity.

**Funding:**

None.

**Research in context**

**Evidence before this study**

We searched PubMed for articles published from 1998 to 2024, with the Medical Subject Heading terms (“non-invasive ventilation”, “high-flow nasal oxygen”, “extubation failure”, “prevention”) and either the supplementary concept (“obese”) or (“obesity”). In a post-hoc analysis of a randomized controlled trial (RCT) of patients after cardiothoracic surgery, including 272 patients with obesity, NIV was not superior to HFNC. In two recent large-scale RCTs including 1098 patients with obesity, no difference in short-term reintubation rates was found between NIV and HFNC.

**Added value of this study**

This network meta-analysis is the most comprehensive synthesis of the available evidence on the use of noninvasive respiratory strategies after extubation in patients with obesity. Across moderate-to-high quality RCTs, this study suggests that NIV with and without HFNC reduces reintubation at day 7 in critically ill patients with obesity, when compared to COT and HFNC. Compared to HFNC, NIV alone or with HFNC reduces mortality. The number needed to treat with NIV or NIV + HFNC to avoid one death was 15.

**Implications of all the available evidence**

This systematic review and network meta-analysis suggests that a systematic use of NIV strategies after extubation reduces extubation failure and mortality in patients with obesity compared to HFNC. These findings support the use of NIV following extubation of critically ill adults with obesity.

## Introduction

Global obesity, defined by a Body Mass Index (BMI) ≥ 30 kg/m^2^, is projected to affect one billion individuals worldwide by the year 2030 according to a report by the World Obesity Federation.^1^ Obesity is a known risk factor for chronic and acute diseases.^2^^,^^3^ In ICU, patients with obesity may undergo invasive mechanical ventilation. After solving the conditions that led to invasive mechanical ventilation, the process of weaning takes place until extubation. Critically ill patients with obesity are considered at high risk after extubation, as many of these patients encounter extubation failure, leading to reintubation, thus increasing the risk of hospital-acquired pneumonia and mortality.^2^^,^^4^

Consequently, optimizing patient management after extubation is paramount in this specific population of critically ill patients with obesity. To this end, several noninvasive respiratory support therapies, including conventional oxygen therapy (COT), noninvasive ventilation (NIV), and more recently high flow nasal cannula (HFNC), have been evaluated to prevent extubation failure in patients with obesity.^5^^,^^6^ However, the literature remains scarce, and large randomized controlled trials (RCTs) did not specifically target patients with obesity until recently. Two large-scale RCTs, involving 1098 patients, have been conducted recently.^5^^,^^7^ A large French multicenter RCT performed on 981 patients with obesity compared COT, HFNC, NIV, and NIV + HFNC.^5^ It reported no difference in reintubation at day 3 in the intention-to-treat analysis but a lower rate in the NIV group in the per-protocol analysis. A Spanish multicenter RCT performed on 144 patients with obesity found no difference in reintubation at day 7 between NIV and HFNC.^7^ The literature being inconclusive, robust guidelines have not yet emerged.^8^^,^^9^

We designed this systematic review and network meta-analysis of RCTs to assess the relative efficacy of COT, NIV, HFNC, and NIV + HFNC after extubation in reducing reintubation of critically ill adult patients with obesity. We hypothesized that NIV and alternating NIV + HFNC may reduce the incidence of reintubation compared to COT or HFNC alone. We also aimed to assess the effect of these strategies on mortality, ICU and hospital length of stay, and atelectasis.

## Methods

We conducted a systematic review and network meta-analysis of RCTs between noninvasive respiratory support therapies (NIV, HFNC, COT, or combination of these), in accordance with the Preferred Reporting Items for Systematic Review and Meta-Analysis (PRISMA) statement extension for network meta-analysis (Supplemental).^10^^,^^11^ The protocol for this systematic review was registered on the PROSPERO register of systematic reviews (CRD42022308995).

### Search strategy and selection criteria

We performed a computerized search of MEDLINE, Cochrane Center Register of Controlled Trials (CENTRAL), and Web of Science databases from 1 January 1998 to 1 July 2024 for RCTs comparing noninvasive respiratory support therapies (NIV, HFNC, COT, or combination of these, either in preventive or curative setting) in which reintubation of critically ill adults with obesity was reported. Studies performed on animals, children, and simulation studies were excluded. No language restriction was applied. We searched abstracts of conferences from 1998 to 2024, including the American Society of Anesthesiologists, the Canadian Anesthesiologists' Society, the International Anesthesia Research Society, the American Thoracic Society, the European Society of Anesthesiology/European Society of Anaesthesiology and Intensive Care, the European Society of Intensive Care Medicine, the European Respiratory Society, the “Société Française d'Anesthésie Réanimation” and the “Société de Réanimation de Langue Française”.^12^ We performed a grey literature search, screening the reference lists of included studies and subsequent guidelines on noninvasive ventilation.

For literature searching, keywords (“Noninvasive ventilation”, “BIPAP”, “CPAP, “High-flow nasal cannula” and “HFNC”) and medical subject headings (“Obesity”, “adult”) were used in our search strategy (Figure S1). Two authors (JP, ANL) screened for relevant RCTs that enrolled adult patients with obesity after extubation undergoing strategies to avoid reintubation. We identified and deleted any duplicate papers. All potential eligible papers were retrieved in full. Then we performed a quantitative synthesis.^9^

### Data analysis

The main outcome was reintubation at day 7.^9^^,^^13^^,^^14^ We selected reintubation at day 7 when several time points were evaluated (n = 5, if not available, reintubation in ICU was used [n = 1], then reintubation at any point [n = 1]). The secondary outcomes were mortality (28-day mortality was selected when available [n = 3], then ICU mortality [n = 2], then mortality at any point [n = 1]),^15^ ICU and hospital length of stay, and atelectasis.

First, two authors (JP, ANL) independently screened the studies by title and abstract for exclusion. They assessed the full text of the possible relevant studies for inclusion and exclusion criteria using a Standardized Data Extraction Sheet (Table S1). Data were added to an Excel database, specifically designed. Disagreement was resolved by discussion and arbitrated by a third author (ADJ).^15^

Data were extracted as they were reported in the original paper or based on the answers of the authors to our queries. Four queries were sent to the authors. Included studies were appraised for their risk of bias by two independent authors (JP, ANL) using the Cochrane Collaboration's tool for RCTs.^16^ Only intention-to-treat estimates from each RCT were extracted. We calculated risk ratios (RR) and 95% confidence intervals (CIs). We performed a pairwise meta-analysis using a Restricted Maximum-Likelihood Estimator random-effects model for all comparisons. We assessed heterogeneity using visual inspection of forest plots, the I^2^ statistic, and the χ^2^ test. We evaluated the feasibility of conducting network meta-analysis by: (1) availability of evidence (number of trials, number of interventions); (2) homogeneity of study designs, patients, and interventions across the body of evidence (transitivity assumption); (3) structural properties of the network of evidence (connectivity); and (4) coherence in network (using the ‘design-by-treatment’ model), and in each closed loop of the network.^9^^,^^13^

We performed a frequentist random-effects network meta-analysis using multivariate meta-analysis assuming a common heterogeneity parameter.^9^^,^^16^ We assessed overall p-values for inconsistency (between direct and indirect comparisons). Then, we used the side-splitting method to assess incoherence between direct and indirect estimates of the effect for each comparison. We estimated ranking probabilities, the Surface Under the Cumulative RAnking Curve (SUCRA), and generated mean treatment rankings.^9^^,^^13^^,^^14^ We conducted analyses using R software (version 4.2.2).

We performed a pre-planned sensitivity pairwise random-effects meta-analysis on reintubation and mortality, comparing NIV and NIV + HFNC (NIV strategies) to HFNC and COT (Oxygen strategies). Prediction intervals were calculated. Prediction intervals in meta-analysis assess the range within which the true effect size of an intervention is expected to fall in future studies, considering the observed heterogeneity across the studies included in the analysis. We used Trial Sequential Analysis to assess the risk of random errors due to sparse data and multiple testing of accumulating data, and to calculate the required information size for reintubation (TSA viewer version 0.9.5.10 Beta).^17^ The calculated required information size considers the control event proportion, the anticipated heterogeneity variance (D^2^) of the meta-analysis, and the assumption of a plausible relative risk reduction (RRR) or relative risk increase (RRI). We used an alpha risk of 5%, a beta risk of 10%, and a D^2^ as suggested by the trials in the meta-analysis.^18^ We used a realistic a priori RRR or RRI of 20%.^15^ We used the Lan-DeMets implementation of the O'Brien-Fleming function to produce the boundaries.^18^ We also performed post-hoc sensitivity random-effects meta-regressions to assess the effect of the baseline reintubation rate on the effect of NIV strategies.^18^

To further explore heterogeneity, post-hoc sensitivity analyses were performed according to the first results reported, especially on studies performed on preventive interventions. A funnel plot was also created to determine the presence of publication bias and other possible biases.^16^

Absolute risk difference was calculated for each comparison, and the number needed to treat (NNT) with its 95% CI was calculated for statistically significant results. All tests were two-sided and p-values less than 0.05 were considered statistically significant.

### Role of the funding source

There was no funding source for this study.

## Results

### Study selection

**Fig. 1:**
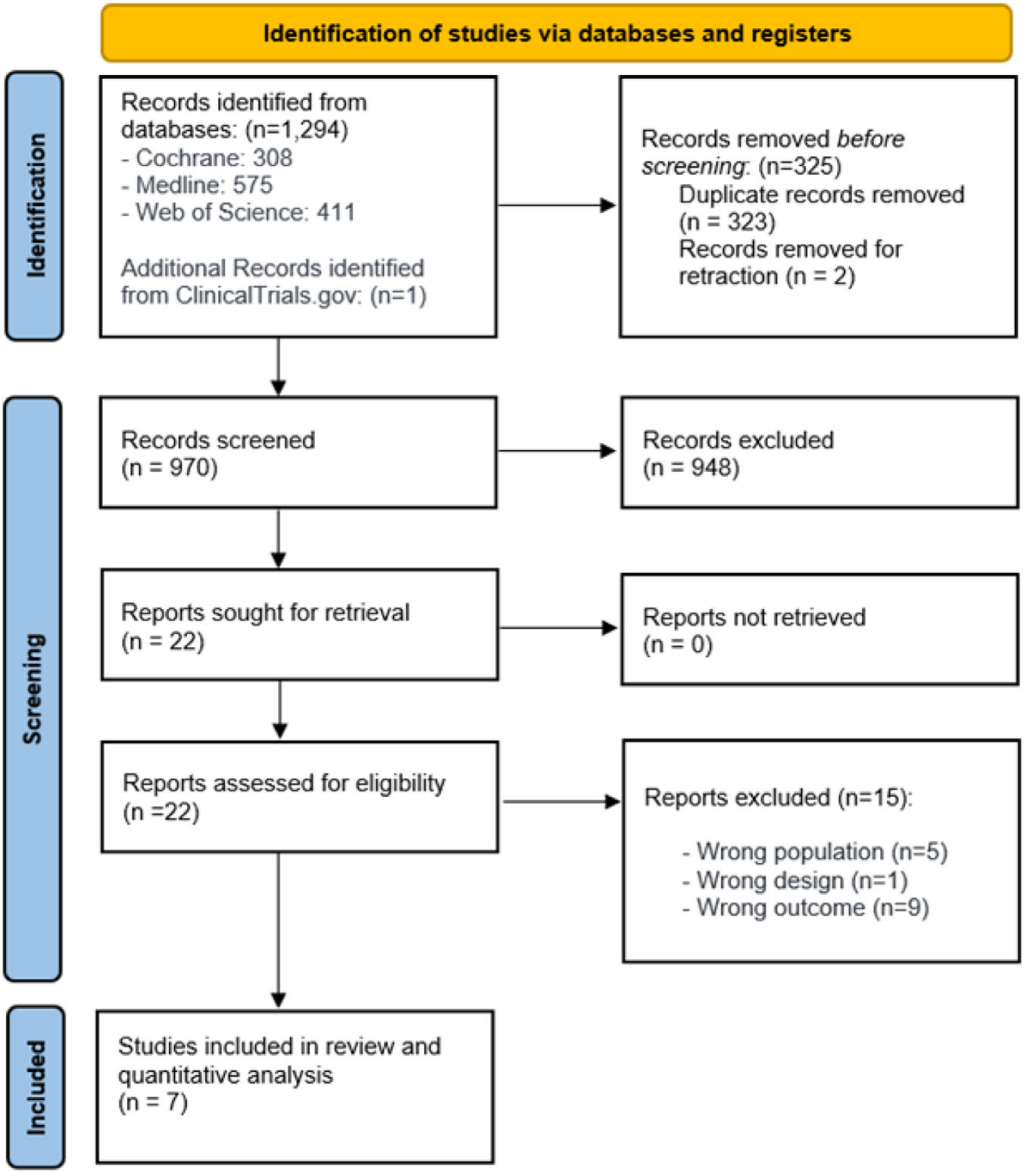
**Flow chart summarizing evidence search and study selection**. WOB, Web of science.

We initially identified 1294 articles using the search strategy (Figure S1), and one additional record through ClinicalTrials.gov.^7^ After excluding 325 citations due to duplications or retraction, and 948 citations on the initial abstract screen because inclusion criteria were not met, we then examined the full-text of the 22 selected papers. We included seven RCTs for the network meta-analysis.^5^, ^6^, ^7^^,^^19^, ^20^, ^21^, ^22^ Fig. 1 shows the study selection flowchart.

### Study description

The seven studies involved a total of 1931 participants from four countries (France n = 4, Spain n = 1, Turkey n = 1, Australia n = 1).^5^, ^6^, ^7^^,^^19^, ^20^, ^21^, ^22^ Characteristics of these studies are presented in Table S2. Among them, 410 patients (21.3% of the patients) received COT (nasal prongs or facemask), 671 patients (34.8%) received HFNC, 495 patients (25.7%) received NIV (facemask interface), and 357 patients (18.2%) received NIV + HFNC. All authors’ queries were answered (Supplemental).^5^, ^6^, ^7^^,^^20^

### Risk of bias assessment

We assessed all RCTs to have a low-to-moderate risk of bias (Table S3).^16^ All trials were assumed to have an intermediate risk of bias regarding blinding.

### Primary outcome: reintubation at day 7

**Fig. 2:**
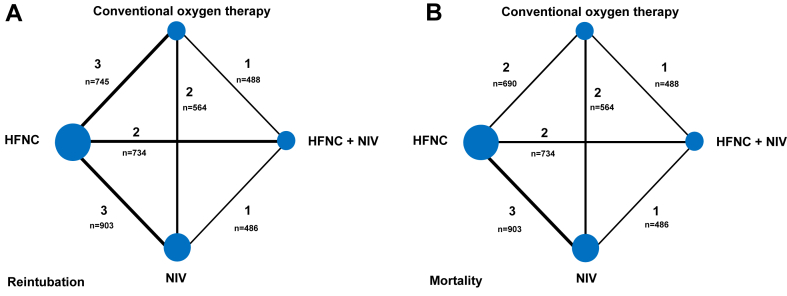
**Network plots for reintubation at day 7 (A) and 28-day mortality (B)**. The size of the node corresponds to the number of patients randomized to that intervention. The thickness of the line and the associated numbers correspond to the number of studies comparing the two linked interventions. The “n” is the number of patients randomized in each comparison. HFNC, High flow nasal cannula; NIV, Noninvasive positive pressure ventilation.

The network plot for reintubation at day 7 is shown in Fig. 2A. The network was dense and well-connected, as each intervention is directly compared with at least one other intervention. The summary of findings, including network estimates, is presented in Table 1. In comparison to COT, NIV + HFNC (RR 0.36 [95% CI 0.16–0.82], high certainty) and NIV (RR 0.45 [95% CI 0.23–0.88], high certainty) significantly reduced reintubation at day 7. However, HFNC (RR 0.79 [95% CI 0.40–1.59], low certainty) did not significantly reduce reintubation at day 7, compared to COT. Compared to HFNC, NIV + HFNC (RR 0.46 [95% CI 0.23–0.90], high certainty) significantly reduced reintubation at day 7. There was no significant difference for NIV compared to HFNC (RR 0.57 [95% CI 0.32–1.02], very low certainty) on reintubation at day 7. The overall p-value for inconsistency was 0.04.

**Table 1:** Network estimates and absolute estimates evaluating the efficacy of the interventions for prevention of reintubation at day 7 in critically ill adults with obesity.

Comparison	Network risk ratio (95% CI)	p-value	Absolute risk difference (95% CI)	Number needed to treat	Grade
NIV vs COT	0.45 (0.23; 0.88)	0.02	−9.3 (−13.0 to −2.0)	11 (8–50)	Moderate^a^
HFNC vs COT	0.79 (0.40; 1.59)	0.51	−3.6 (−10.2 to 9.9)	NA	Low^a^^,^^b^
NIV vs HFNC	0.57 (0.32; 1.02)	0.06	−5.8 (−9.1 to 0.3)	NA	Very low^a^^,^^b^^,^^c^
NIV + HFNC vs COT	0.36 (0.16; 0.82)	0.01	−10.8 (−14.2 to −3.0)	10 (7–33)	Moderate^a^
NIV + HFNC vs NIV	0.80 (0.38; 1.72)	0.57	−2.9 (−9.1 to 10.7)	NA	Very low^a^^,^^b^^,^^c^
NIV + HFNC vs HFNC	0.46 (0.23; 0.90)	0.02	−7.2 (−10.3 to −1.3)	14 (10–77)	Moderate^a^

NIV, Noninvasive positive pressure ventilation; HFNC, High flow nasal cannula; COT, Conventional oxygen therapy.

a Lowered one level for risk of bias.

b Lowered one level for imprecision as CIs don't exclude harm.

c Lowered for inconsistency.

Direct estimates, indirect estimates, and SUCRA table are provided in Table S4. NIV + HFNC had a 53.3% chance of being the best strategy, compared to 46.4% for NIV, 0.2% for HFNC, and 0.1% for COT. The funnel plot is shown on Figure S2.

In a sensitivity analysis performed on preventive strategies, NIV + HFNC reduced significantly reintubation at day 7 (RR 0.33 [95% CI 0.12–0.95], moderate certainty) compared to COT. The network plot and the summary of findings are presented in Table S5. Direct estimates, indirect estimates, and SUCRA table are provided in Table S6. The funnel plot is shown on Figure S3. The overall p-value for inconsistency was 0.08.

**Fig. 3:**
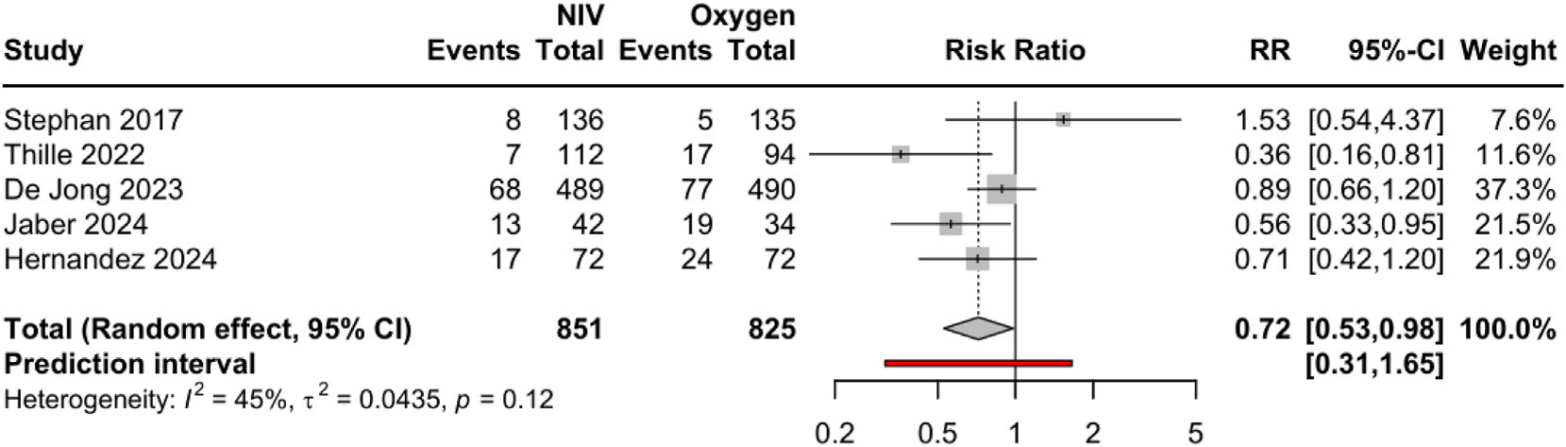
**Forest plot of NIV strategies on reintubation at day 7 compared to Oxygen strategies in critically ill patients with obesity**. NIV strategies: NIV or NIV + HFNC. Oxygen strategies: HFNC or COT. NIV: Noninvasive positive pressure ventilation, HFNC, High flow nasal cannula; COT, Conventional oxygen therapy.

In a sensitivity pairwise meta-analysis, NIV and NIV + HFNC (NIV strategies) were compared to HFNC and COT (Oxygen strategies). Five studies and 1676 patients were included in this analysis.^5^, ^6^, ^7^^,^^20^^,^^21^ In random effect, the pooled RR across all studies was 0.72 (95% CI 0.53–0.98), indicating a significant reduction of 7-day reintubation with NIV strategies compared to Oxygen strategies (Fig. 3). The funnel plot is shown on Figure S4. In Trial Sequential Analysis, the trial sequential monitoring boundaries for benefit, harm, or futility were not crossed by the Z-curve (Figure S5). The required information size was estimated to be 4043. In meta-regression, the baseline reintubation rate did not significantly moderate the effect of NIV on reintubation (p = 0.11, Figure S6). Figures S7 and S8 show the sensitivity pairwise meta-analysis performed on preventive strategies.

### Secondary outcomes

#### 28-Day mortality

The network plot for 28-day mortality is shown in Fig. 2B. The network was dense and well-connected, as each intervention is directly compared with at least one other intervention. The summary of findings, including network estimates, is presented in Table 2. In comparison to COT, neither NIV + HFNC (RR 0.40 [95% CI 0.11–1.43], low certainty), NIV (RR 0.41 [95% CI 0.13–1.25], low certainty) nor HFNC (RR 1.32 [95% CI 0.43–4.10], very low certainty) showed a significant reduction of 28-day mortality. Compared to HFNC, both NIV + HFNC (RR 0.30 [95% CI 0.10–0.89], high certainty) and NIV alone (RR 0.31 [95% CI 0.13–0.74], moderate certainty) significantly reduced 28-day mortality. The overall p-value for inconsistency was 0.02.

**Table 2:** Network estimates and absolute estimates evaluating the efficacy of the interventions for prevention of 28-day mortality in critically ill adults with obesity.

Comparison	Network risk ratio (95% CI)	p-value	Absolute risk difference (95% CI)	Number needed to treat	Grade
NIV vs COT	0.41 (0.13; 1.25)	0.12	−9.6 (−14.1 to 4.1)	NA	Low^a^^,^^b^
HFNC vs COT	1.32 (0.43; 4.10)	0.63	5.2 (−9.2 to 50.4)	NA	Very low^a^^,^^b^^,^^c^
NIV vs HFNC	0.31 (0.13; 0.74)	<0.01	−6.6 (−8.3 to −2.5)	15 (12–40)	Low^a^^,^^c^
NIV + HFNC vs COT	0.40 (0.11; 1.43)	0.16	−9.8 (−14.5 to 7.0)	NA	Low^a^^,^^b^
NIV + HFNC vs NIV	0.97 (0.29; 3.19)	0.96	−0.3 (−6.3 to 19.5)	NA	Very low^a^^,^^b^^,^^c^
NIV + HFNC vs HFNC	0.30 (0.10; 0.89)	0.03	−6.7 (−8.6 to −1.1)	15 (11–90)	Moderate^a^

NIV, Noninvasive positive pressure ventilation; HFNC, High flow nasal cannula; COT, Conventional oxygen therapy.

a Lowered one level for risk of bias.

b Lowered one level for imprecision as CIs don't exclude harm.

c Lowered for inconsistency.

Direct estimates, indirect estimates, and SUCRA table are provided in STable 7. NIV had a 65.1% chance of being the best strategy, followed by 27.7% for NIV + HFNC, 7.2% for HFNC, and 0.0% for COT. The funnel plot is shown on Figure S9.

In a sensitivity analysis performed on preventive strategies, both NIV + HFNC (RR 0.31 [95% CI 0.10–0.96], moderate certainty) and NIV alone (RR 0.28 [95% CI 0.11–0.72], moderate certainty) significantly reduced 28-day mortality compared to HFNC. The network plot and the summary of findings are presented in Table S8. The overall p-value for inconsistency was 0.02. Direct estimates, indirect estimates, and SUCRA table are displayed in Table S9. The funnel plot is shown on Figure S10.

In a sensitivity pairwise meta-analysis, the NIV strategies were compared to the Oxygen strategies. Five studies and 1676 patients were included in this analysis.^5^, ^6^, ^7^^,^^20^^,^^21^ In random effect, the pooled RR across all studies was 0.81 (95% CI 0.48–1.35), indicating no significant reduction of 28-day mortality with NIV strategies compared to Oxygen strategies (Figure S11). The funnel plot is shown on Figure S12. In meta-regression, the baseline reintubation rate did not significantly moderate the effect of NIV on mortality (p = 0.23, Figure S13).

#### ICU length of stay

The network plot and the summary of findings are presented in Table S10. None of the strategies was associated with a significant reduction in ICU length of stay across all comparisons. The overall p-value for inconsistency was 0.94. Direct estimates, indirect estimates, and SUCRA table are shown in Table S11. The funnel plot is shown on Figure S14.

In a sensitivity analysis performed on preventive strategies, none of the strategies was associated with a significant reduction in ICU length of stay across all comparisons. The overall p-value for inconsistency was 0.99. The network plot and the summary of findings are presented in Table S12. Direct estimates, indirect estimates, and SUCRA table are provided in Table S13. The funnel plot is shown on Figure S15.

#### Hospital length of stay

The network plot and the summary of findings are presented in Table S14. None of the strategies was associated with a significant reduction in hospital length of stay across all comparisons. The overall p-value for inconsistency was 0.15. Direct estimates, indirect estimates, and SUCRA table are shown in Table S15. The funnel plot is shown on Figure S16.

In a sensitivity analysis performed on preventive strategies, none of the strategies was associated with a significant reduction in hospital length of stay across all comparisons. The network plot and the summary of findings are presented in Table S16. The overall p-value for inconsistency was 0.08. Direct estimates, indirect estimates, and SUCRA table are provided in Table S17. The funnel plot is shown on Figure S17.

#### Atelectasis

Both studies that presented results for this outcome compared HFNC and COT, using the Radiological Atelectasis Score (RAS). Compared to COT, HFNC did not reduce atelectasis (mean difference in RAS –0.40 [95% CI –1.20 to 0.41], low certainty, Tables S18 and S19).

## Discussion

The present network meta-analysis suggests that following extubation of critically ill patients with obesity, NIV alone or in combination with HFNC may reduce the risk of reintubation compared to COT (Moderate confidence). Compared to HFNC, the combination of NIV and HFNC may also reduce the risk of reintubation (Moderate confidence). NIV-based strategies may reduce the risk of reintubation when compared to Oxygen-based strategies. This study also found evidence that NIV alone or in combination with HFNC may reduce mortality compared to HFNC (Low to Moderate confidence). Overall, the probability of being the best for both NIV and the combination of NIV and HFNC reached 99.7% regarding reintubation and 92.8% regarding mortality in this study.

Recent network meta-analyses have been performed either on overall critically ill adults after extubation,^9^ on overall critically ill adults with acute respiratory failure after extubation,^13^ or on medical critically ill patients after extubation.^14^ These works found that NIV and HFNC might be superior to COT in preventing reintubation. However, no significant difference was found between NIV and HFNC in either of these studies.^9^^,^^13^^,^^14^ Meanwhile, the European Society of Intensive Care Medicine guidelines provided conditional recommendation with low certainty for HFNC in high-risk patients who had received invasive ventilation for more than 24 h.^8^ In patients with obesity, previous observational studies have reported that clinicians use preferentially NIV after extubation, with the aim to mitigate reintubation.^23^ However, individual RCTs have struggled to identify a significant superiority of NIV over HFNC or COT.^5^^,^^7^ For the first time with a such level of evidence, the current study highlights the superiority of NIV strategies over HFNC and COT to mitigate reintubation and mortality.

The discrepancies between these results could be explained by the specific needs of critically ill patients with obesity.^24^^,^^25^ Our results suggest that, in critically ill patients with obesity, providing positive pressure might be the cornerstone to prevent extubation failure. Specifically, positive pressure as provided by NIV seems to be required in this patient population, for both atelectasis and sleep-related breathing disorders.^24^ In comparison, HFNC does not provide sufficient positive pressure, and COT provides no positive pressure. Those findings are consistent with the main respiratory physiological modifications induced by obesity, which lead to shunt via atelectasis and gas exchange impairment: decreased functional residual capacity, increased abdominal pressure, decreased pulmonary and chest wall compliance, cephalic ascension of the diaphragm, and increased oxygen consumption and work of breathing.^23^^,^^26^, ^27^, ^28^ However, whether NIV sessions should be associated with HFNC or COT between the sessions is still unclear. As only one study compared directly the two strategies,^5^ large confidence intervals prevent to draw any conclusion regarding this comparison. Further research is needed between those two interventions, especially as they emerge as the two best interventions throughout our study.

Among the seven included studies, only one (the NIVAS study)^20^ reported the effects of NIV in patients with acute respiratory failure after extubation. In the other six studies,^5^, ^6^, ^7^^,^^19^^,^^21^^,^^22^ interventions were applied as prophylactic strategies in end to mitigate reintubation after extubation. However, the sensitivity analyses performed on preventive support strategies after extubation did not modify the main message (Figure S7, Tables S5 and S6). Three studies included high-risk overall critically ill patients and four included postoperative critically ill patients (Table S2). Interventions were applied for at least 24–48 h across the studies. All NIV protocols included bilevel positive airway pressure, but the minimal duration of application varied widely, from 4 to 12 h per day. HFNC protocols were broadly similar, with a set flow after the initiation period between 40 and 60 L/min if tolerated (Table S2). The most common outcome definition for reintubation was 7-day (used in 5/7 studies). For mortality, the most frequent time points were 28-day (3/6 studies) and ICU mortality (2/6 studies).

The main strength of this study is that it focuses on noninvasive respiratory support in critically ill patients with obesity, which lowers the clinical heterogeneity between studies and highlights the peculiarities of patients with obesity. The study included a substantial number of patients, augmented by recent RCTs dedicated to patients with obesity. Sensitivity analyses were consistent with the main analyses, enhancing the reliability of the findings. The network format enabled the comparison of multiple strategies and their combinations, providing a comprehensive understanding of the available evidence.^9^^,^^16^

Our systematic review and meta-analysis have several limitations. First of all, three of the included trials were post-hoc analyses of RCTs including non-obese patients.^4^^,^^29^^,^^30^ Second, statistical heterogeneity and inconsistency were observed in some analyses, although we performed multiple sensitivity analyses. The results of network meta-analyses may potentially be influenced by indirect evidence.^9^^,^^16^ However, in this review, we did not find issues with intransitivity, and the network estimates were largely driven by direct data, with coherent indirect data. Moreover, the inclusion of both preventive and curative interventions causes clinical heterogeneity, with different reintubation rates between studies. Thus, we performed separate sensitivity analyses, which stratified preventive interventions, leading to similar results, even if meta-regressions may lack of statistical power. Third, variability in NIV and HFNC protocols, including pressure and flow levels, was noted among the included studies. Then, blinding of the participants was not feasible due to the nature of the interventions. Moreover, some individual results may appear paradoxical and difficult to read for physicians, as many results fall at the limit between statistical significance and unsignificance. This might reflect either a lack of power in some comparisons, or heterogeneity and inconsistency between studies. The results of the Bayesian SUCRA estimation may be difficult to align with the frequentist network meta-analysis results. The aim of the SUCRA estimation is to give an overview of the results that is closer to the clinical practice, by ranking the interventions rather than interpreting numerous comparisons. The simplest message of the SUCRA analysis in our study might be summed up as: NIV and NIV + HFNC are probably the interventions to be chosen to mitigate reintubation and mortality.

The results of the present systematic review and network meta-analysis suggest that NIV, alone or in combination with HFNC, is may be superior to COT and HFNC to prevent reintubation after extubation in critically ill patients with obesity. NIV and NIV + HFNC may be superior to HFNC to prevent mortality. The number needed to treat with NIV or NIV + HFNC to avoid one death was 15. These findings support the proactive implementation of NIV after extubation of critically ill adults with obesity, with a moderate level of confidence. Focus should be given to identifying other categories of patients who might benefit from NIV after extubation.

## Contributors

SJ contributed to the conception and the design of the study, to the acquisition of the data, to drafting the submitted article and to provide final approval of the version to be published. JP contributed to conception and design of the study, to the acquisition of the data, to the analysis of the data, to drafting the submitted article, and to provide final approval of the version to be published. ANL contributed to conception and design of the study, to the acquisition of the data, to the analysis of the data, to drafting the submitted article, and to provide final approval of the version to be published. ADJ contributed to the conception and design of the study, to the analysis and interpretation of data, to drafting the submitted article, and to provide final approval of the version to be published. NM contributed to the analysis and interpretation of data, to drafting the submitted article, and to provide final approval of the version to be published. SJ, JP, ANL, ADJ, and NM accessed and verified the data. CM, MC, YA, IL, and GC contributed to the interpretation of data and to provide final approval of the version to be published. All authors provide agreement to be accountable for all aspects of the work in ensuring that questions related to the accuracy or integrity of any part of the work are appropriately investigated and resolved.

## Data sharing statement

Research data and other material will be made available to the scientific community, immediately on publication, with as few restrictions as possible. All requests should be submitted to the corresponding author who will review with the other investigators for consideration. A data use agreement will be required before the release of participant data and institutional review board approval as appropriate.

## Declaration of interests

SJ reports receiving consulting fees from Drager, Medtronic, Baxter, Fresenius, Xenios, Mindray, and Fisher & Paykel. ADJ reports receiving consulting fees from Medtronic, Viatris, Sanofi and Sedana. JP reports receiving a Research Grant from the “Société Française d’Anesthésie-Réanimation”. No conflict of interests is reported for other authors.
