# Supplementary material for: Noninvasive respiratory support following extubation in critically ill adults with obesity: a systematic review and network meta-analysis
Source: eClinicalMedicine. 2024 Dec 16;79:103002. doi: 10.1016/j.eclinm.2024.103002 (PMC11715126; doi:10.1016/j.eclinm.2024.103002)
Supplement: Supplemental [file mmc1.docx]

**SUPPLEMENTARY MATERIAL**

- **Supplemental Figure 1.** Electronic Search Strategies.
- **Supplemental Figure 2.** Funnel plot for reintubation at day 7.
- **Supplemental Figure 3.** Funnel plot for reintubation at day 7 in a sensitivity analysis on preventive support.
- **Supplemental Figure 4.** Funnel plot for reintubation at day 7 in meta-analysis comparing noninvasive positive pressure ventilation to oxygen therapy alone.
- **Supplemental Figure 5.** Trial sequential analysis for reintubation at day 7 comparing noninvasive positive pressure ventilation to oxygen therapy alone.
- **Supplemental Figure 6.** Meta-regression on the effect of baseline reintubation rate on the effect of noninvasive positive pressure ventilation on reintubation at day 7.
- **Supplemental Figure 7.** Forest plot for reintubation at day 7 in meta-analysis comparing noninvasive positive pressure ventilation to oxygen therapy alone, sensitivity analysis on preventive support.
- **Supplemental Figure 8.** Funnel plot for reintubation at day 7 in meta-analysis comparing noninvasive positive pressure ventilation to oxygen therapy alone, sensitivity analysis on preventive support.
- **Supplemental Figure 9.** Funnel plot for 28-day mortality.
- **Supplemental Figure 10.** Funnel plot for 28-day mortality in a sensitivity analysis on preventive support.
- **Supplemental Figure 11.** Forest plot for 28-day mortality in meta-analysis comparing noninvasive positive pressure ventilation to oxygen therapy alone.
- **Supplemental Figure 12.** Funnel plot for 28-day mortality in meta-analysis comparing noninvasive positive pressure ventilation to oxygen therapy alone.
- **Supplemental Figure 13.** Meta-regression on the effect of baseline reintubation rate on the effect of noninvasive positive pressure ventilation on 28-day mortality.
- **Supplemental Figure 14.** Funnel plot for ICU length of stay.
- **Supplemental Figure 15.** Funnel plot for ICU length of stay in a sensitivity analysis on preventive support.
- **Supplemental Figure 16.** Funnel plot for Hospital length of stay.
- **Supplemental Figure 17.** Funnel plot for Hospital length of stay in a sensitivity analysis on preventive support.
- **Supplemental Table 1.** Standardized Data Extraction Sheet.
- **Supplemental Table 2.** Detailed Characteristics of the 7 Included Studies, and Number of Patients Randomized to Included Therapies.
- **Supplemental Table 3.** Quality Assessment for Risk of Bias of the 7 studies.
- **Supplemental Table 4.** Direct and indirect estimates, tests of coherence, and Surface Under the Cumulative Ranking curve (SUCRA) for reintubation at day 7 in critically ill adults.
- **Supplemental Table 5.** Network diagram, network estimates and absolute estimates evaluating the efficacy of the interventions for prevention of reintubation at day 7 in critically ill adults in a sensitivity analysis on preventive support.
- **Supplemental Table 6.** Direct and indirect estimates, tests of coherence, and Surface Under the Cumulative Ranking curve (SUCRA) for reintubation at day 7 in critically ill adults in a sensitivity analysis on preventive support.
- **Supplemental Table 7.** Direct and indirect estimates, tests of coherence, and Surface Under the Cumulative Ranking curve (SUCRA) for 28-day mortality in critically ill adults.
- **Supplemental Table 8.** Network and absolute estimates evaluating the efficacy of the interventions for prevention of 28-day mortality in critically ill adults in a sensitivity analysis on preventive support.
- **Supplemental Table 9.** Direct and indirect estimates, tests of coherence, and Surface Under the Cumulative Ranking curve (SUCRA) for 28-day mortality in critically ill adults in a sensitivity analysis on preventive support.
- **Supplemental Table 10.** Network diagram, network estimates and absolute estimates evaluating the efficacy of the interventions for ICU length of stay in critically ill adults.
- **Supplemental Table 11.** Direct and indirect estimates, tests of coherence, and SUCRA table evaluating the efficacy of the interventions for ICU length of stay.
- **Supplemental Table 12.** Network diagram, network estimates and absolute estimates evaluating the efficacy of the interventions for ICU length of stay in critically ill adults in a sensitivity analysis on preventive support.
- **Supplemental Table 13.** Direct and indirect estimates, tests of coherence, and SUCRA table evaluating the efficacy of the interventions for ICU length of stay in a sensitivity analysis on preventive support.
- **Supplemental Table 14.** Network diagram and network estimates evaluating the efficacy of the interventions for the Hospital length of stay in critically ill adults.
- **Supplemental Table 15.** Direct and indirect estimates, tests of coherence, and SUCRA table evaluating the efficacy of the interventions for Hospital length of stay.
- **Supplemental Table 16.** Network diagram and network estimates evaluating the efficacy of the interventions for the Hospital length of stay in critically ill adults in a sensitivity analysis on preventive support.
- **Supplemental Table 17.** Direct and indirect estimates, tests of coherence, and SUCRA table evaluating the efficacy of the interventions for Hospital length of stay.
- **Supplemental Table 18.** Network diagram and network estimates evaluating the efficacy of the interventions for the Radiological Atelectasis Score (RAS) in critically ill.
- **Supplemental Table 19.** Direct and indirect estimates, tests of coherence, and SUCRA table evaluating the efficacy of the interventions for Radiological Atelectasis Score (RAS).
- **Results obtained from queries to the authors**

# Supplemental Figure 1: Electronic Search Strategies.

**Databases Searched:**

- PubMed/Medline
- Web of Science
- Cochrane Central Register of Controlled Trials (CENTRAL)

**PubMed/MEDLINE**

|  | extubation,,,"""airway extubation""[MeSH Terms] OR (""airway""[All Fields] AND ""extubation""[All Fields]) OR ""airway extubation""[All Fields] OR ""extubated""[All Fields] OR ""extubation""[All Fields] OR ""extubations""[All Fields] OR ""extubate""[All Fields] OR ""extubating""[All Fields]","16,631",07:01:10AND ""extubation""[All Fields]) OR ""airway extubation""[All Fields] OR ""extubated""[All Fields] OR ""extubation""[All Fields] OR ""extubations""[All Fields] OR ""extubate""[All Fields] OR ""extubating""[All Fields]", | 16,631 |
| --- | --- | --- |
|  | wean*,,,"""wean*""[All Fields]" | 59,985 |
| 3. | postoperative,,,"""postoperative period""[MeSH Terms] OR (""postoperative""[All Fields] AND ""period""[All Fields]) OR ""postoperative period""[All Fields] OR ""postop""[All Fields] OR ""postoperative""[All Fields] OR ""postoperatively""[All Fields] OR ""postoperatives""[All Fields]" | 1,017,342 |
| 4. | 4,#1 OR #2 OR #3,,,"""airway extubation""[MeSH Terms] OR (""airway""[All Fields] AND ""extubation""[All Fields]) OR ""airway extubation""[All Fields] OR ""extubated""[All Fields] OR ""extubation""[All Fields] OR ""extubations""[All Fields] OR ""extubate""[All Fields] OR ""extubating""[All Fields] OR ""wean*""[All Fields] OR (""postoperative period""[MeSH Terms] OR (""postoperative""[All Fields] AND ""period""[All Fields]) OR ""postoperative period""[All Fields] OR ""postop""[All Fields] OR ""postoperative""[All Fields] OR ""postoperatively""[All Fields] OR ""postoperatives""[All Fields])" | 1,081,892 |
| 5. | nasal cannu*,,,"(""nasalance""[All Fields] OR ""nasality""[All Fields] OR ""nasalization""[All Fields] OR ""nasalized""[All Fields] OR ""nasally""[All Fields] OR ""nose""[MeSH Terms] OR ""nose""[All Fields] OR ""nasal""[All Fields] OR ""nasals""[All Fields]) AND ""cannu*""[All Fields]" | 3,816 |
| 6. | nasal oxygen,,,"(""nasalance""[All Fields] OR ""nasality""[All Fields] OR ""nasalization""[All Fields] OR ""nasalized""[All Fields] OR ""nasally""[All Fields] OR ""nose""[MeSH Terms] OR ""nose""[All Fields] OR ""nasal""[All Fields] OR ""nasals""[All Fields]) AND (""cell respiration""[MeSH Terms] OR (""cell""[All Fields] AND ""respiration""[All Fields]) OR ""cell respiration""[All Fields] OR ""oxygenation""[All Fields] OR ""oxygen""[MeSH Terms] OR ""oxygen""[All Fields] OR ""oxygen s""[All Fields] OR ""oxygenate""[All Fields] OR ""oxygenated""[All Fields] OR ""oxygenates""[All Fields] OR ""oxygenating""[All Fields] OR ""oxygenations""[All Fields] OR ""oxygenative""[All Fields] OR ""oxygenator s""[All Fields] OR ""oxygenators""[MeSH Terms] OR ""oxygenators""[All Fields] OR ""oxygenator""[All Fields] OR ""oxygene""[All Fields] OR ""oxygenic""[All Fields] OR ""oxygenous""[All Fields] OR ""oxygens""[All Fields])" | 8,302 |
| 7. | noninvasive ventilation,,,"""noninvasive ventilation""[MeSH Terms] OR (""noninvasive""[All Fields] AND ""ventilation""[All Fields]) OR ""noninvasive ventilation""[All Fields]" | 13,928 |
| 8. | noninvasive ventilation[tw],,,"""noninvasive ventilation""[Text Word]" | 6,140 |
| 9. | positive pressure ventilation,,,"""positive pressure respiration""[MeSH Terms] OR (""positive pressure""[All Fields] AND ""respiration""[All Fields]) OR ""positive pressure respiration""[All Fields] OR (""positive""[All Fields] AND ""pressure""[All Fields] AND ""ventilation""[All Fields]) OR ""positive pressure ventilation""[All Fields] OR ""intermittent positive pressure ventilation""[MeSH Terms] OR (""intermittent""[All Fields] AND ""positive pressure""[All Fields] AND ""ventilation""[All Fields]) OR ""intermittent positive pressure ventilation""[All Fields] OR (""positive""[All Fields] AND ""pressure""[All Fields] AND ""ventilation""[All Fields])" | 37,008 |
| 10. | BIPAP,,,"""continuous positive airway pressure""[MeSH Terms] OR (""continuous""[All Fields] AND ""positive""[All Fields] AND ""airway""[All Fields] AND ""pressure""[All Fields]) OR ""continuous positive airway pressure""[All Fields] OR ""bipap""[All Fields]" | 15,984 |
| 11. | face mask,,,"(""face""[MeSH Terms] OR ""face""[All Fields]) AND (""masks""[MeSH Terms] OR ""masks""[All Fields] OR ""mask""[All Fields])" | 8,668 |
| 12. | oxygen therapy,,,"""oxygen inhalation therapy""[MeSH Terms] OR (""oxygen""[All Fields] AND ""inhalation""[All Fields] AND ""therapy""[All Fields]) OR ""oxygen inhalation therapy""[All Fields] OR (""oxygen""[All Fields] AND ""therapy""[All Fields]) OR ""oxygen therapy""[All Fields]" | 141,393 |
| 13. | #5 OR #6 OR #7 OR #8 OR #9 OR #10 OR #11 OR #12 | 193,523 |
| 14. | obes*,,,"""obes*""[All Fields]" | 455,901 |
| 15. | obesity,,,"""obeses""[All Fields] OR ""obesity""[MeSH Terms] OR ""obesity""[All Fields] OR ""obese""[All Fields] OR ""obesities""[All Fields] OR ""obesity s""[All Fields]" | 439,207 |
| 16. | BMI,,,"""BMI""[All Fields]" | 186,914 |
| 17. | overweight,,,"""overweight""[MeSH Terms] OR ""overweight""[All Fields] OR ""overweighted""[All Fields] OR ""overweightness""[All Fields] OR ""overweights""[All Fields]" | 296,224 |
| 18. | #14 OR #15 OR #16 OR #17 | 570,096 |
| 19. | #4 AND #13 AND #18 | 575 |

**Cochrane Central Register of Controlled Trials (CENTRAL)**

| 1. | (extubation OR "wean*" OR postoperative) AND ("obes*" OR 'BMI OR overweight OR Obesity) AND ("noninvasive ventilation" OR positive pressure ventilation OR "BIPAP" OR "nasal cannu*" OR "face mask" OR "oxygen therapy" OR CPAP OR nasal oxygen) (Word variations have been searched) | 364 |
| --- | --- | --- |

**Web of Science**

| 1. | extubation OR "wean*" OR postoperative  (All Fields) AND "obes*" OR 'BMI OR overweight OR Obesity  (All Fields) AND "noninvasive ventilation" OR positive pressure ventilation OR "BIPAP" OR "nasal cannu*" OR "face mask" OR "oxygen therapy" OR CPAP OR nasal oxygen  (All Fields) | 411 |
| --- | --- | --- |

**Supplemental Figure 2:** Funnel plot for 7-day reintubation.

**
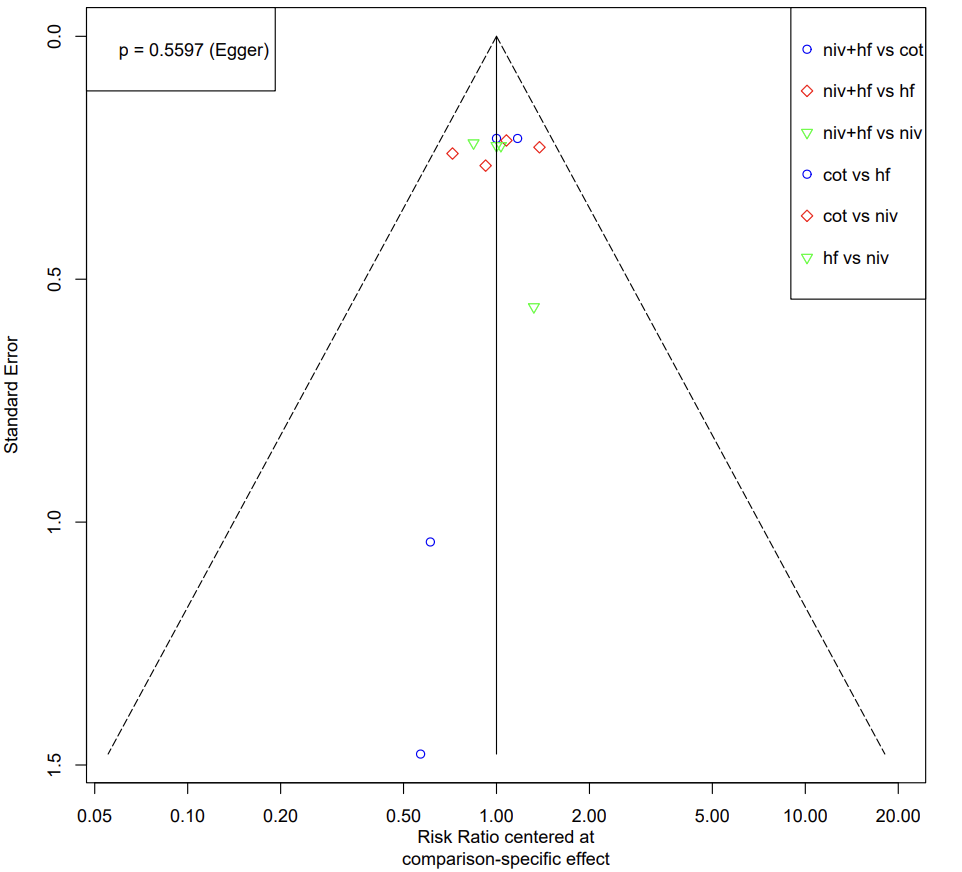
**

**Supplemental Figure 3:** Funnel plot for 7-day reintubation in a sensitivity analysis on preventive support.


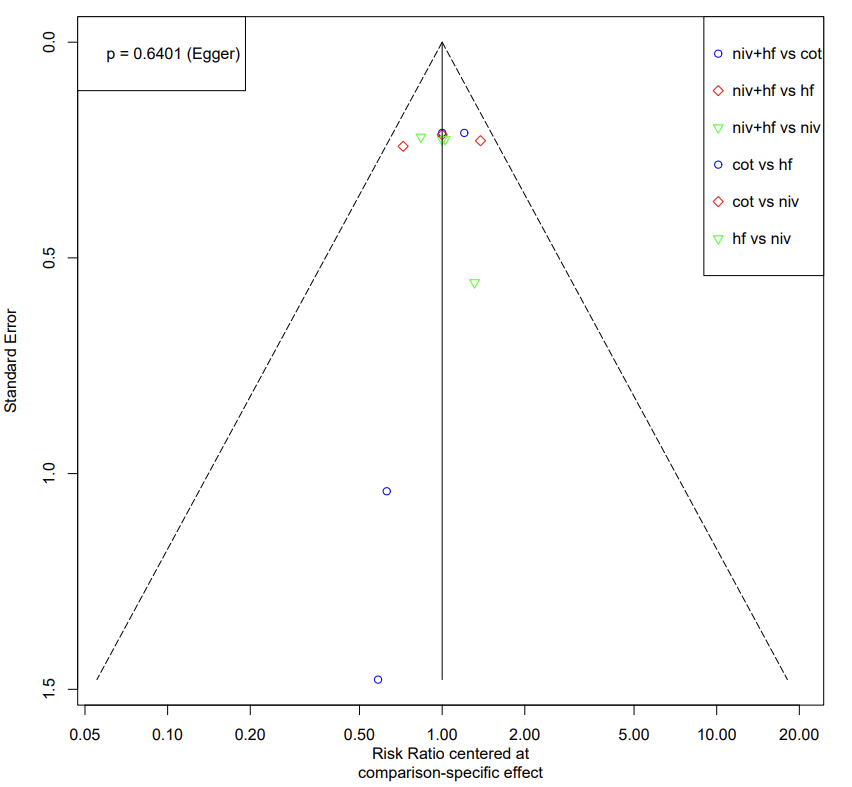


**Supplemental Figure 4:** Funnel plot for 7-day reintubation in meta-analysis comparing noninvasive positive pressure ventilation to oxygen therapy alone.

P-value for Egger test = 0.63.


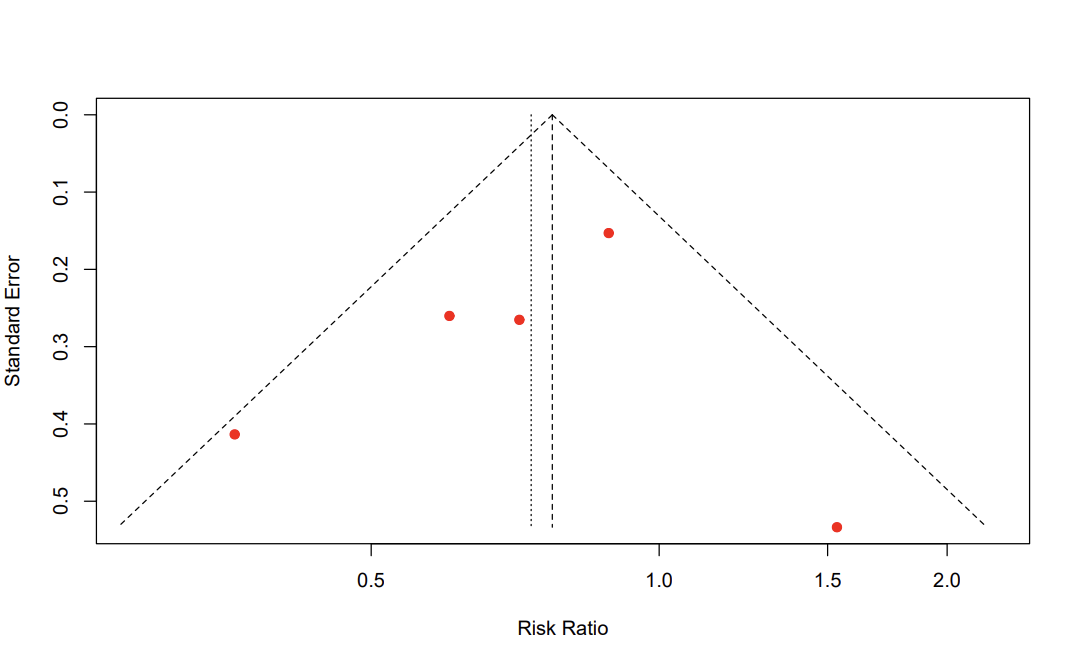


**Supplemental Figure 5:** Trial sequential analysis for 7-day reintubation comparing noninvasive positive pressure ventilation to oxygen therapy alone.


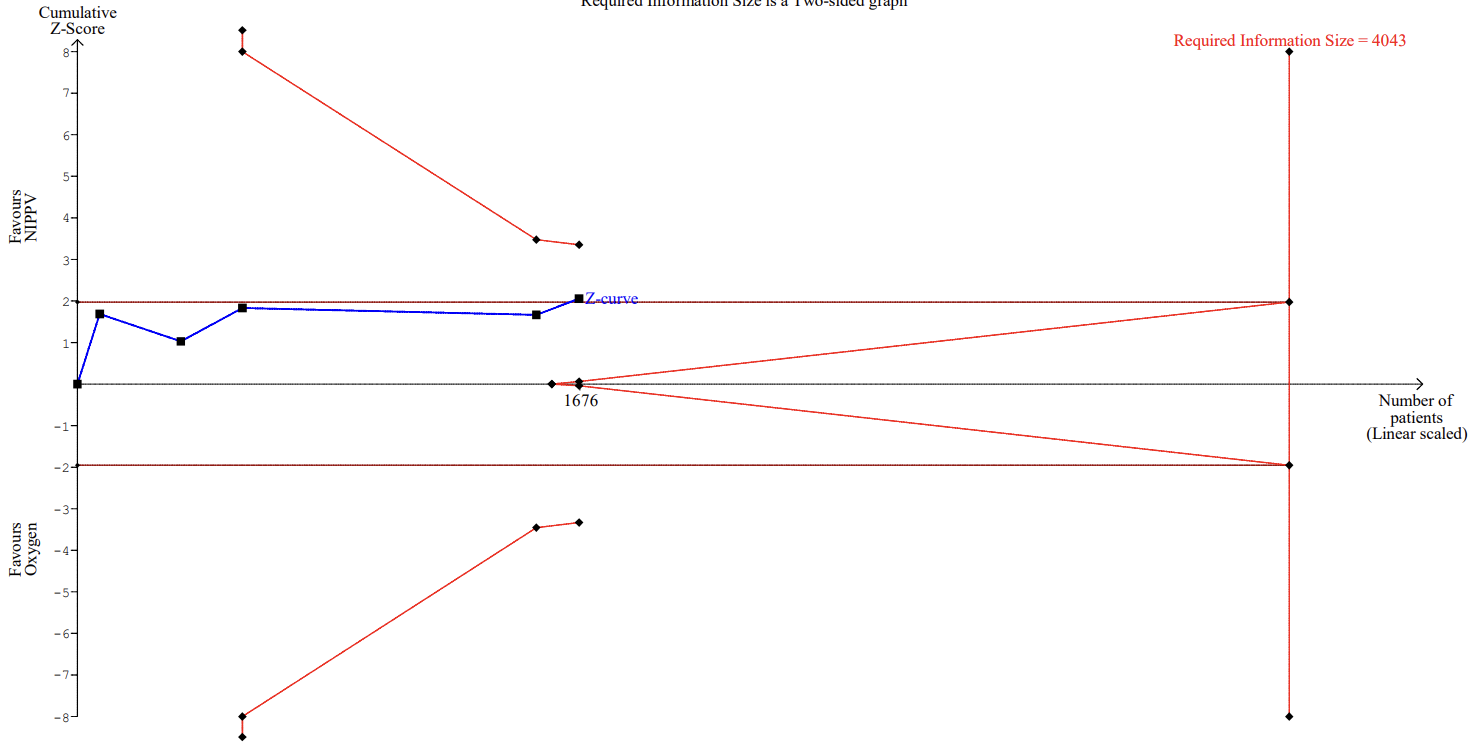


**Supplemental Figure 6.** Meta-regression on the effect of baseline reintubation rate on the effect of noninvasive positive pressure ventilation on reintubation at day 7.


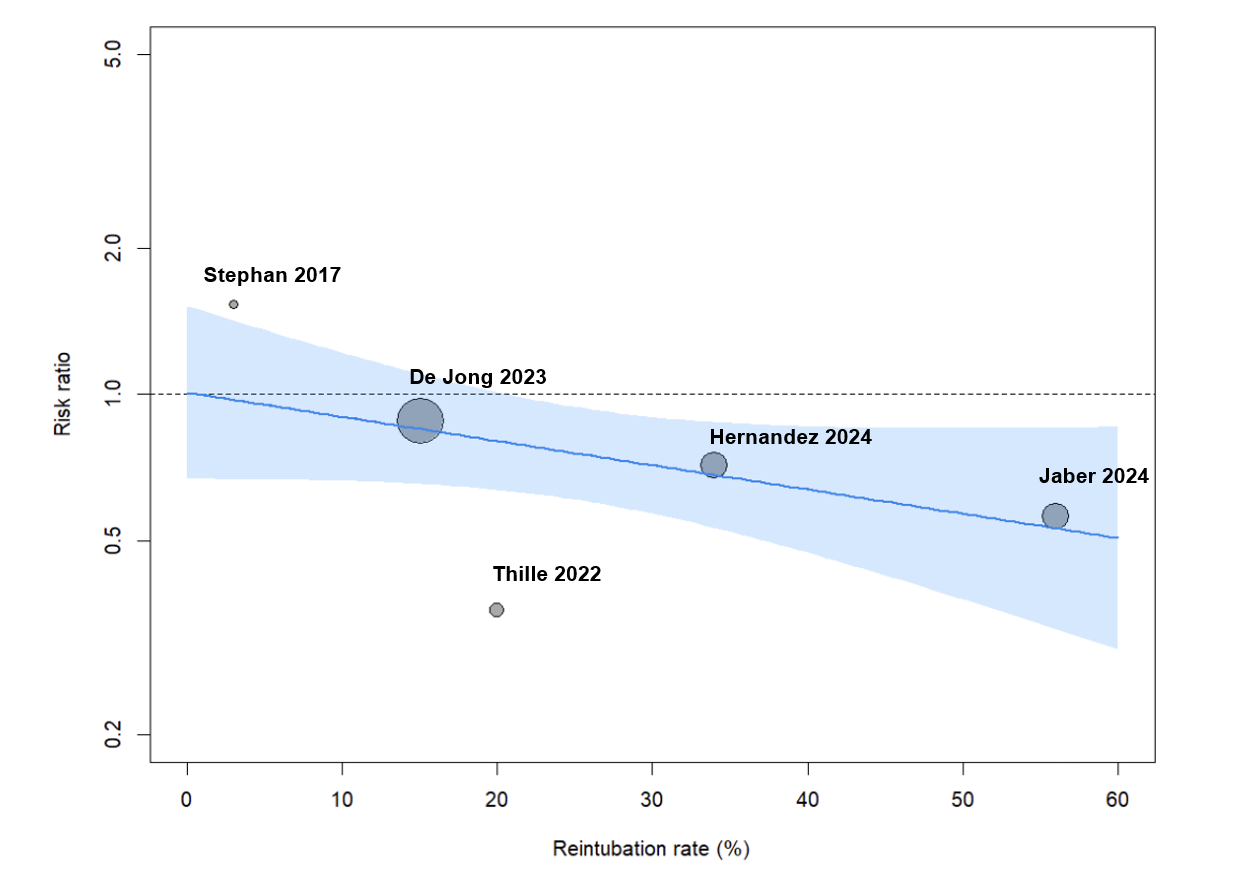


RR = 0·89 [95%CI 0·78-1·02] per 10% increase of the baseline reintubation rate, p= 0·10

I2 = 0%, p-value for residual heterogeneity: 0·20

**Supplemental Figure 7:** Forest plot for 7-day reintubation in meta-analysis comparing noninvasive positive pressure ventilation to oxygen therapy alone, sensitivity analysis on preventive support.

**
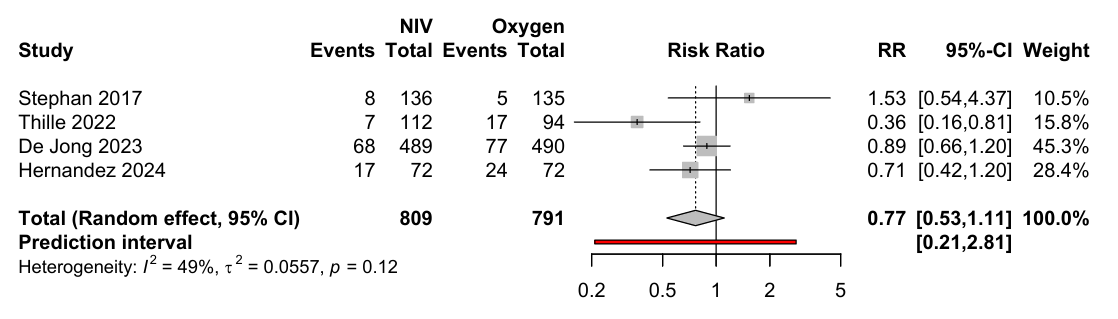
**

**Supplemental Figure 8:** Funnel plot for reintubation at day 7 in meta-analysis comparing noninvasive positive pressure ventilation to oxygen therapy alone, sensitivity analysis on preventive support.

P-value for Egger test = 0.74

**
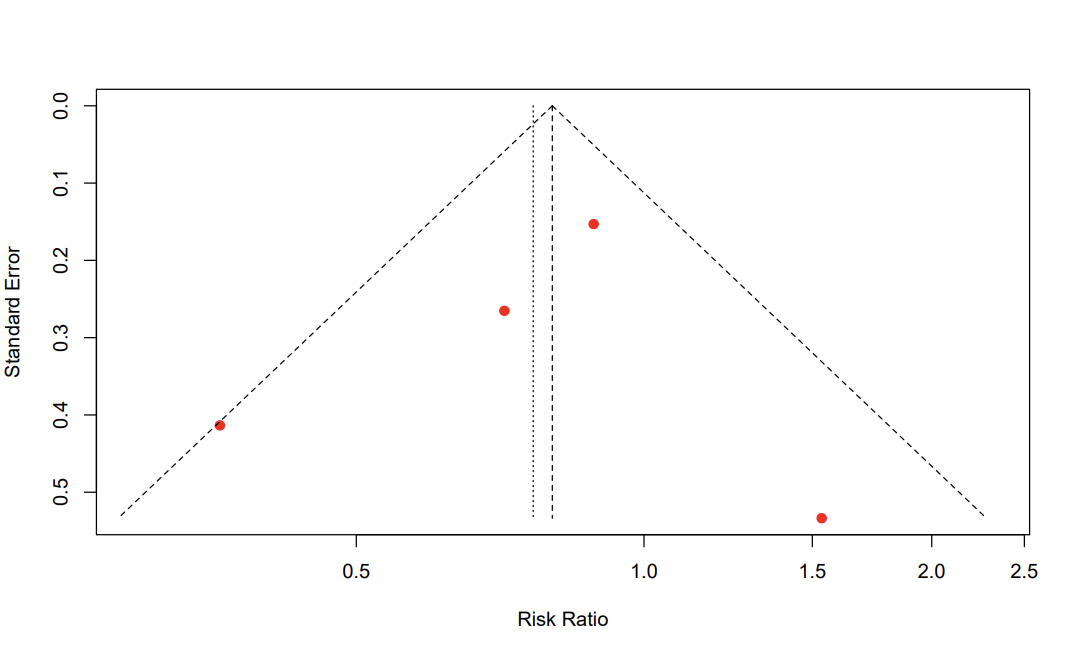
**

**Supplemental Figure 9:** Funnel plot for 28-day mortality.


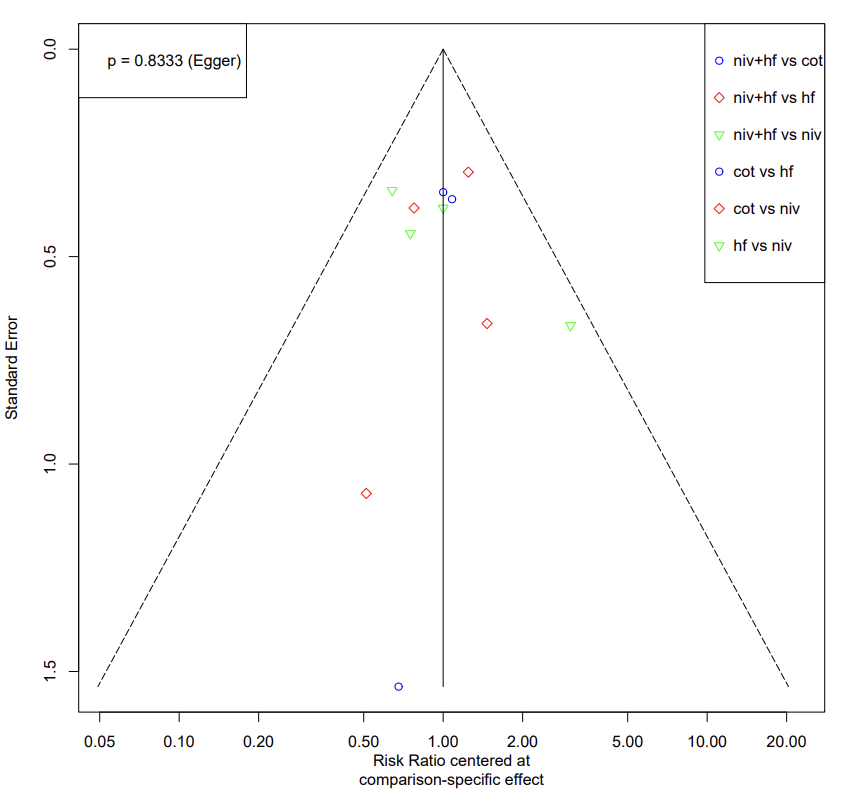


**Supplemental Figure 10:** Funnel plot for 28-day mortality in a sensitivity analysis on preventive support.


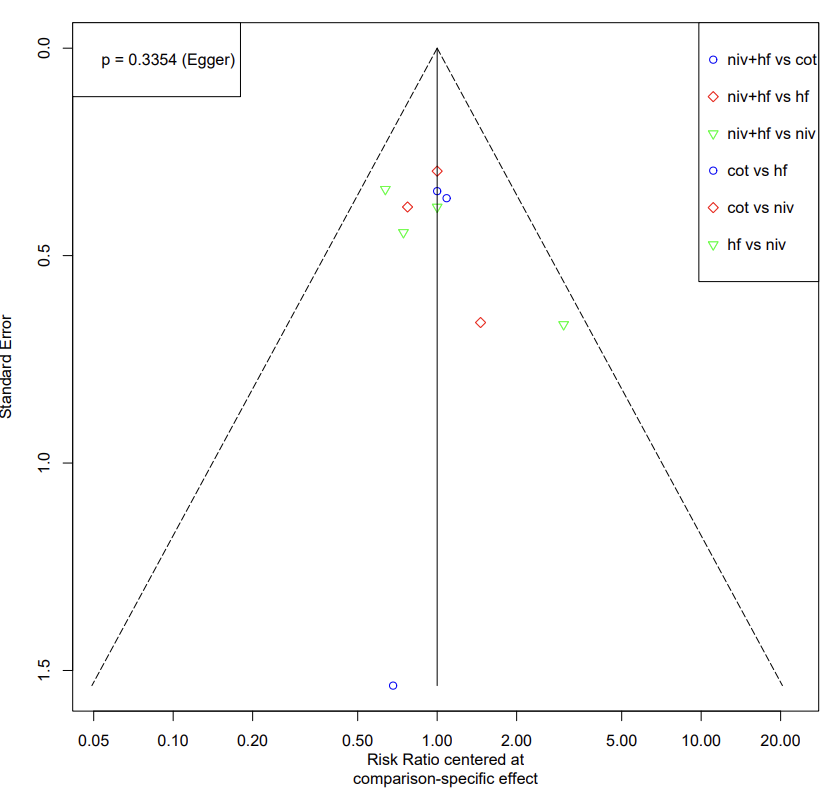


**Supplemental Figure 11:** Forest plot for 28-day mortality in meta-analysis comparing noninvasive positive pressure ventilation to oxygen.


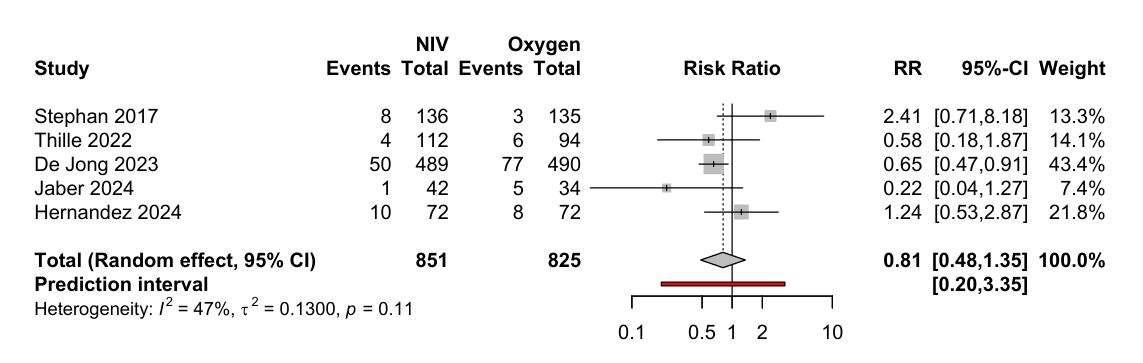


**Supplemental Figure 12:** Funnel plot for 28-day mortality in meta-analysis comparing noninvasive positive pressure ventilation to oxygen therapy alone.

P-value for Egger test = 0.71.

**
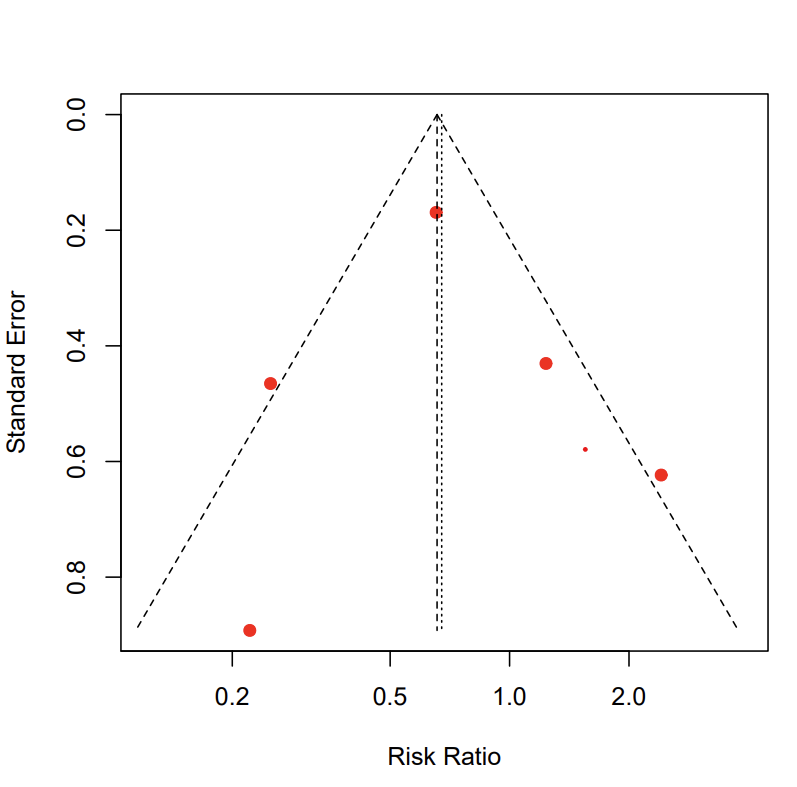
**

**Supplemental Figure 13.** Meta-regression on the effect of baseline reintubation rate on the effect of noninvasive positive pressure ventilation on 28-day mortality.

**
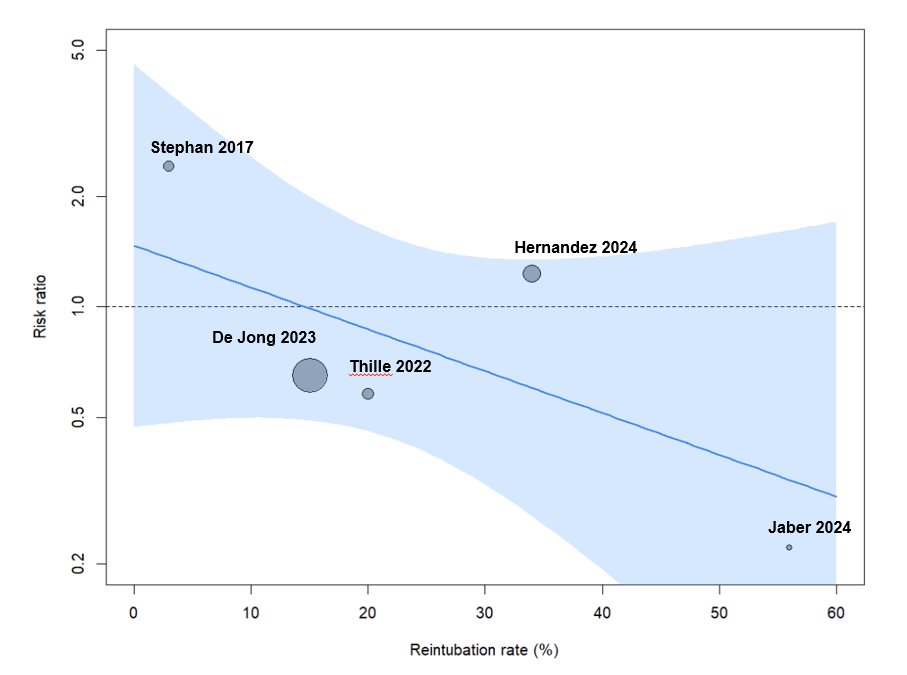
**

RR = 0·77 [95%CI 0·50-1·18] per 10% increase of the baseline reintubation rate, p= 0·23

I2 = 57%, p-value for residual heterogeneity: 0·07

**Supplemental Figure 14:** Funnel plot for ICU length of stay

**
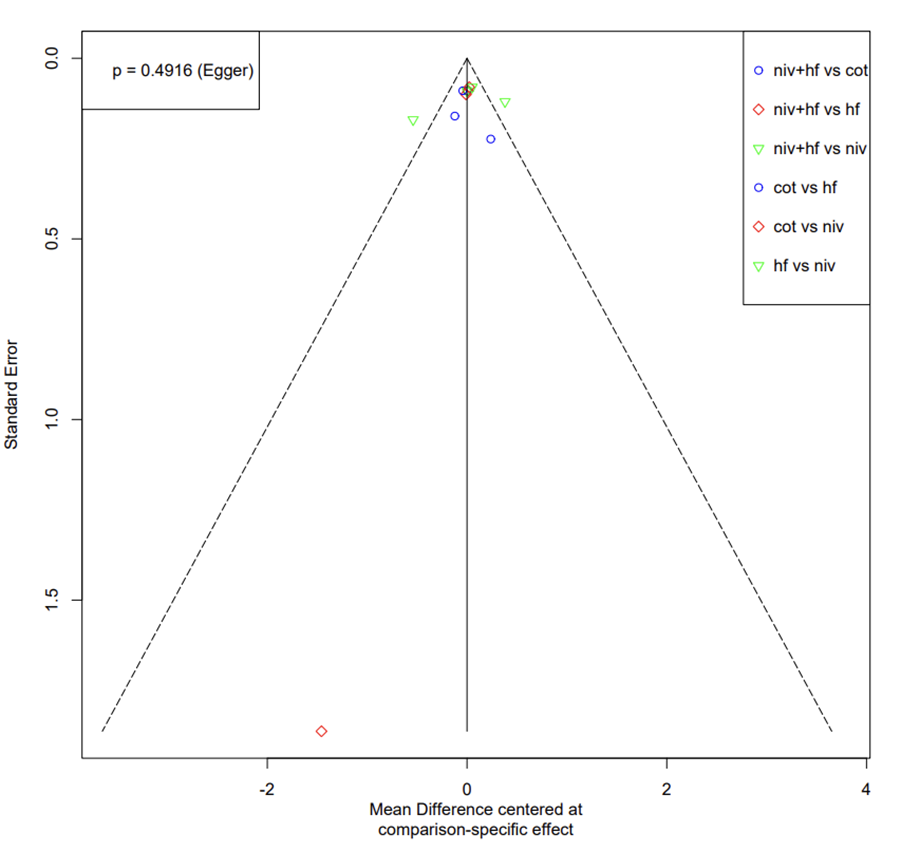
**

**Supplemental Figure 15:** Funnel plot for ICU length of stay in a sensitivity analysis on preventive support.


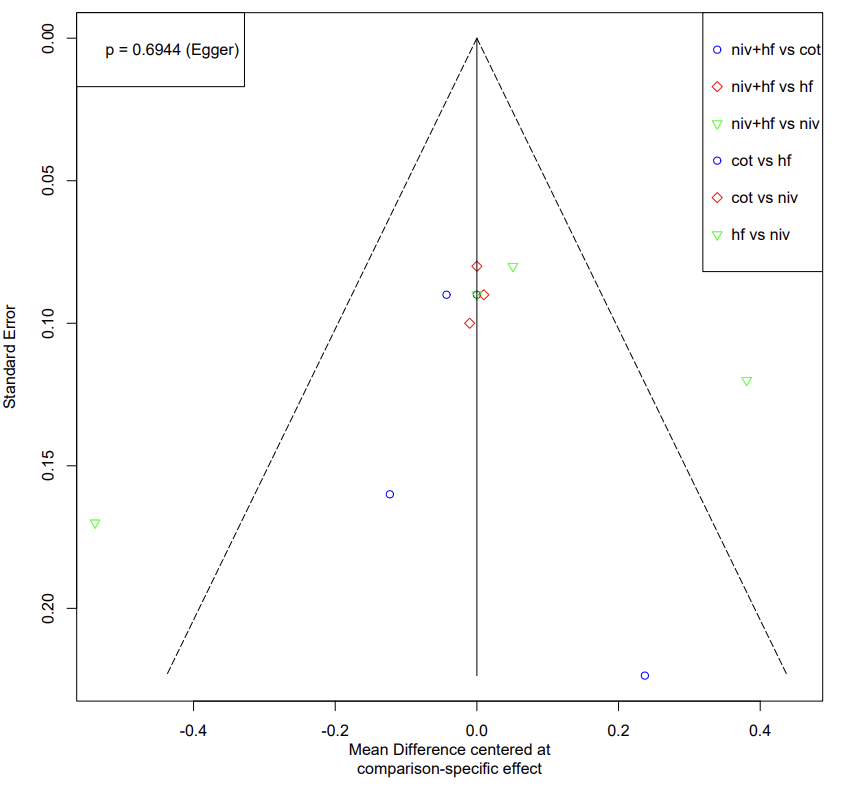


**Supplemental Figure 16:** Funnel plot for Hospital length of stay

**
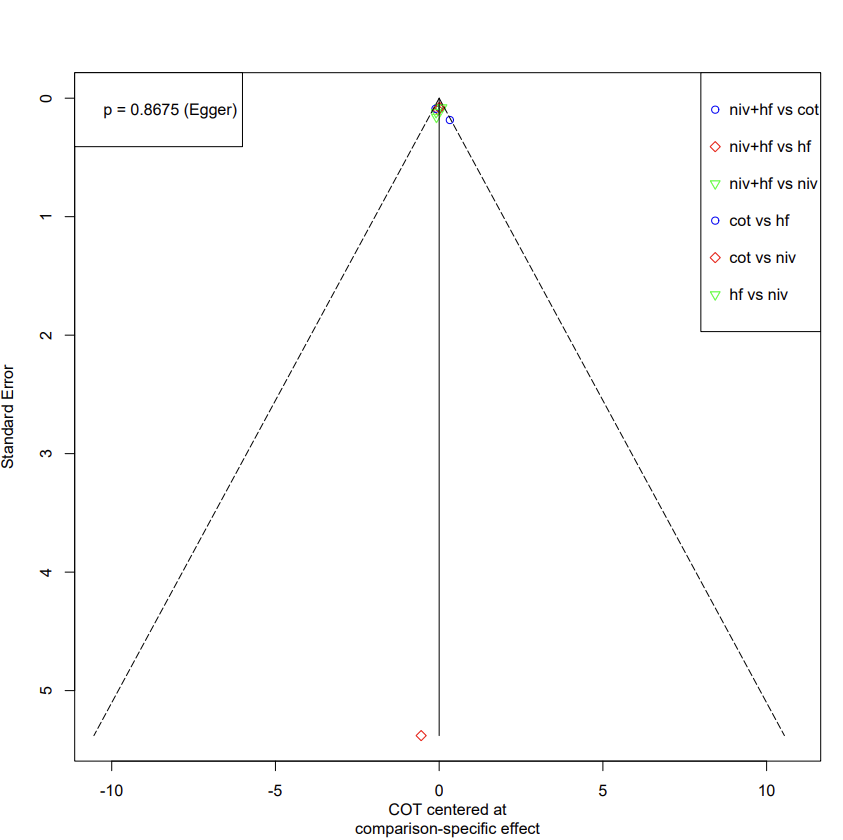
**

**Supplemental Figure 17:** Funnel plot for Hospital length of stay in a sensitivity analysis on preventive support.

**
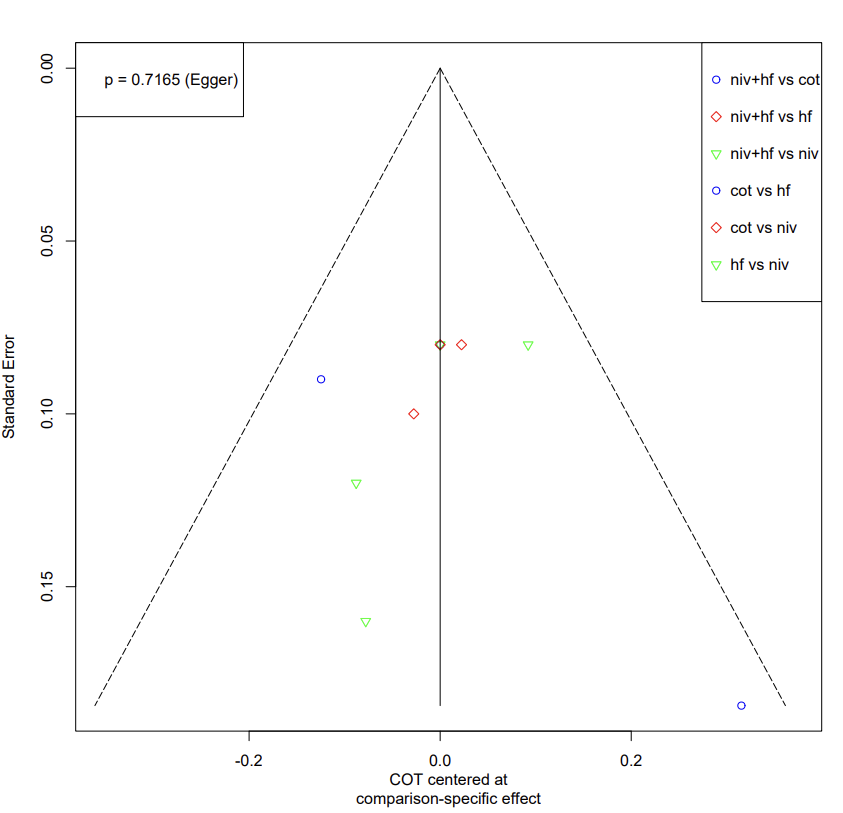
Supplemental Table 1:** Standardized Data Extraction Sheet.

| **Data to be Extracted** | **Notes to Reviewer** |
| --- | --- |
| **Basic Study Information** | |
| Study Title |  |
| Journal/Conference |  |
| Conference Abstract vs. Full-text |  |
| Year of Publication |  |
| Language | If published in language other than English - Exclude |
| Author | List first author only |
| Correspondence Email |  |
| Randomized Trial? | If “No” – Exclude |
| Parallel vs. Crossover vs. Cluster |  |
| Number of Sites |  |
| Country/Countries of Study |  |
| **Eligibility Assessment** | |
| Does the study include adult patients (i.e. ≥ 18 years of age)? | If “No” – Exclude |
| Does the study include patient with obesity (i.e. BMI ≥ 30 ?) | If “No” – Exclude |
| Was the study conducted primarily (i.e. ≥ 70%) in patients admitted to an Intensive Care Unit (ICU)? | If “No” – Exclude |
| Are patients randomized to receive at least one of the following: 1) High-flow nasal cannula (HFNC); 2) Non-invasive positive pressure ventilation (NIV); 3) Conventional oxygen therapy (COT); or 4) A combination of any of the above? | If “No” – Exclude |
| Is the study conducted in the post-extubation setting, and compares the interventions for prevention or rescue? | If “No” – Exclude |
| Are any of the following outcomes evaluated: 1) Re-intubation (at any point during ICU admission); 2) Short-term mortality; 3) Long-term mortality; 3) Time to re-intubation; 4) ICU length of stay; or 5) Hospital length of stay? | If “No” – Exclude |
| Was the trial conducted in the emergency department, operating room, or post-anesthetic care unit? | If “Yes” – Exclude |
| Did the trial evaluate the effect of the interventions on “weaning” (e.g. passing a spontaneous breathing trial), but not provide data specifically on extubation failure? | If “Yes” – Exclude |
| Did the study exclusively evaluate patients with self-extubation? | If “Yes” – Exclude |
| Did the study exclusively enroll patients in the palliative setting? | If “Yes” – Exclude |
| Did the study randomize patients to weaning or post-extubation protocols instead of treatments? | If “Yes” – Exclude |
| **Trial Characteristics** | |
| Was population was included? | E.g. mixed, surgical, hypercapneic, etc. |
| What was the mean duration of hospitalization? |  |
| Were elderly patients included? |  |
| Were pregnant patients included? |  |
| Were patients with any other co-morbidity included/excluded? |  |
| Intervention 1 |  |
| Intervention 2 |  |
| Intervention 3 (if applicable) |  |
| Intervention 4 (if applicable) |  |
| **Risk of Bias Assessment** | |
| How were patients randomized ? |  |
| In either the intervention or the control group, were there any deviations from the protocol ? |  |
| Was there any missing outcome data ? |  |
| Were the outcome measures objective ? |  |
| Were all important pre-specified outcomes presented ? |  |
| Any other bias noted ? |  |
| **Outcome #1** | |
| Outcome being evaluated | e.g. Re-intubation |
| Dichotomous or continuous outcome? |  |
| Intervention 1: N analyzed |  |
| Intervention 1: Number of Events/Mean |  |
| Intervention 2: N analyzed |  |
| Intervention 2: Number of Events/Mean |  |
| Intervention 3: N analyzed |  |
| Intervention 3: Number of Events/Mean |  |
| Intervention 4: N analyzed |  |
| Intervention 4: Number of Events/Mean |  |
| Comments |  |
| **Outcome #2** | |
| Outcome being evaluated | e.g. 30-day mortality |
| Dichotomous or continuous outcome? |  |
| Intervention 1: N analyzed |  |
| Intervention 1: Number of Events/Mean |  |
| Intervention 2: N analyzed |  |
| Intervention 2: Number of Events/Mean |  |
| Intervention 3: N analyzed |  |
| Intervention 3: Number of Events/Mean |  |
| Intervention 4: N analyzed |  |
| Intervention 4: Number of Events/Mean |  |
| Comments |  |
| **Author Contact** | |
| Contact author? | If more information needed, indicate here to contact author |

Supplemental Table 2: Detailed Characteristics of the 7 Included Studies, and Number of Patients Randomized to Included Therapies.

| **Author**  **(Trial Name)** | **Year** | **Journal** | **Centers** | **Country** | **Population** | **Indication for treatment** | **Inclusion Criteria** | **Exclusion Criteria** | **Treatment Arms** | **Duration** | **Outcomes of interest** | ***N*** | **Mean Age** | **% Male** |
| --- | --- | --- | --- | --- | --- | --- | --- | --- | --- | --- | --- | --- | --- | --- |
| Hernández | 2024 | *American Journal of Respiratory and Critical Care Medicine* | 2 | Spain | High-risk critically ill patients | Preventive | BMI≥30kg/m²; 18 years; >48hrs IMV; “≤ 2 “High risk" features (age >65, heart failure, COPD, APACHE II >12, airway patency problems, inability to deal with secretions, difficult or prolonged weaning, ≥2 comorbidities, IMV ≥7 days) | Hypercapnia at the end of SBT, do-not-resuscitate orders, tracheostomies, accidental or self-extubation, contraindication for NIV | 1)NIV  2)HFNC | > 48 hours  NIV > 12 hours per day, up to 24h if tolerated, bilevel  HFNC = 60L/min if tolerated | - 7-day reintubation  - 28-day mortality  - ICU and hospital LOS | 144 | 61 | 45% |
| Jaber | 2024 | *Intensive Care Medicine* | 20 | France | Surgical (abdominal) | Curative | >18 years; abdominal surgery; acute respiratory failure within day 7 | Contraindications to NIV; Withholding life-sustaining treatment; Obstructive sleep apnea; immediate tracheal intubation, Urgent surgery; | 1)NIV  2)COT | > 24 hours  NIV > 6 hours per day, bilevel | - 7-day reintubation  - 28-day mortality  - ICU and hospital LOS | 76 | 63 | 76% |
| De Jong | 2023 | *Lancet Respiratory Medicine* | 39 | France | Critically ill patients | Preventive | BMI≥30kg/m²; >18 years; ICU admission; IMV>6hrs; | Hypercapnia; Isolated cardiogenic pulmonary oedema; Tracheostomy; home ventilation; do not reintubate order; previous extubation | 1) NIV + HFNC  2) NIV + COT  3) HFNC  4) COT | > 24 hours  NIV > 4 hours per day, bilevel  HFNC = 50L/min if tolerated | - 7-day reintubation  - 28-day mortality  - ICU and hospital LOS | 981 | 61 | 61% |
| Thille | 2022 | *American Journal of Respiratory and Critical Care Medicine* | 30 | France | High-risk critically ill patients | Preventive | BMI≥30kg/m²; >18 years; IMV > 24hrs; One "high risk" criteria (>65 years; Underlying chronic cardiac or lung disease) | Long-term treatment with NIV or CPAP at home; Contraindication to NIV; Underlying chronic neuromuscular disease; TBI leading to intubation; Unplanned extubation | 1)NIV + HFNC  2)HFNC | > 48 hours  NIV > 12 hours per day, bilevel  HFNC = 50L/min if tolerated | - 7-day reintubation  - ICU mortality  - ICU and hospital LOS | 206 | 70 | 59% |
| Sahin | 2018 | *Canadian Respiratory Journal* | 1 | Turkey | Surgical (Cardiac) critically ill patients | Preventive | BMI≥30kg/m²; Following Cardiopulmonary Bypass | Hemodynamic instability; <18 years; Tracheostomy; Obstructive sleep apnea; Active pulmonary disease; Urgent surgery; Low cardiac output | 1)HFNC  2) COT | Applied throughout the ICU stay (mean duration >48 hours)  HFNC = 40L/min if tolerated | - ICU reintubation  - Hospital mortality  - ICU and hospital LOS | 100 | 61 | 36% |
| Stephan | 2017 | *Respiratory Care* | 6 | France | Surgical (Cardiac) critically ill patients* | Preventive | BMI≥30kg/m²; Cardiothoracic surgery and any of the following: 1) Failure of SBT; 2) Successful SBT but risk factors for postextubation failure; or 3) Previous failed extubation | Tracheostomy; Do-not-intubate order; Obstructive sleep apnea; Delirium; Nausea and vomiting; Bradypnea; Impaired consciousness; Hemodynamic instability | 1)NIV  2)HFNC | > 24 hours  NIV > 4 hours per day, bilevel  HFNC = 50L/min if tolerated | - 7-day reintubation  - ICU mortality  - ICU and hospital LOS | 271 | 64 | 62% |
| Corley | 2015 | *Intensive Care Medicine* | 1 | Australia | Surgical (Cardiac) critically ill patients | Preventive | BMI≥30kg/m²; >18 years; Cardiac surgery on cardiopulmonary bypass | IMV > 36hrs; Extubation onto NIV; Tracheostomy; Extubation for palliation | 1)HFNC  2)COT | Applied throughout the ICU stay (mean duration >36 hours)  HFNC = 50L/min if tolerated | - 1-day reintubation  - ICU and hospital LOS | 155 | 64 | 73% |

* A *post-hoc* analysis was performed in patients with obesity, the landmark study included both patients with and without obesity

ICU = intensive care unit; IMV = invasive mechanical ventilation; BMI = body mass index; ARF = acute respiratory failure; LOS = length of stay; COPD = chronic obstructive pulmonary disease: APACHE = acute physiology and chronic health evaluation; SBT = spontaneous breathing trial; NIV = noninvasive positive pressure ventilation; COT = conventional oxygen therapy; HFNC = high flow nasal oxygen; VAP = ventilator acquired pneumonia; VAT = ventilator acquired tracheobronchitis; CPAP = continuous positive airway pressure; TBI = traumatic brain injury; ABG = arterial blood gas; FEV1 = forced expiratory volume at 1 second; FVC = forced vital capacity; PaO2/FiO2 = ratio of oxygen arterial pressure under oxygen arterial fraction; PACU = postanesthesia care unit; ASA = American society of anesthesiology score

# Supplemental Table 3: Quality Assessment for Risk of Bias of the 7 studies.

| **Author (Year)** | **Journal** | **Random Sequence Generation** | **Allocation Sequence Concealment** | **Blinding** | **Incomplete Outcome Data** | **Selective reporting** | **Other Bias** |
| --- | --- | --- | --- | --- | --- | --- | --- |
| Hernández 2024 | *American Journal of Respiratory and Critical Care Medicine* | Low | Low | Some concerns | Low | Low | Low |
| Jaber 2024 | *Intensive Care Medicine* | Low | Low | Some concerns | Low | Low | Low |
| De Jong 2023 | *Lancet Respiratory Medicine* | Low | Low | Some concerns | Low | Low | Low |
| Thille 2022 | *American Journal of Respiratory and Critical Care Medicine* | Low | Low | Some concerns | Low | Low | Low |
| Sahin 2018 | *Canadian Respiratory Journal* | Low | Low | Some concerns | Low | Low | Low |
| Stephan 2017 | *Respiratory Care* | Low | Low | Some concerns | Low | Low | Low |
| Corley 2015 | *Intensive Care Medicine* | Low | Low | Some concerns | Low | Low | Low |

Supplemental Table 4: Direct and indirect estimates, tests of coherence, and Surface Under the Cumulative Ranking curve (SUCRA) for 7-day reintubation in critically ill adults.

NIV: Noninvasive positive pressure ventilation, HFNC: High flow nasal oxygen, COT: Conventional oxygen therapy.

| **Comparison** | **Direct RR** | **Indirect RR** | **Network meta-analysis RR** | **Direct evidence proportion** | **P value for incoherence** |
| --- | --- | --- | --- | --- | --- |
| HFNC vs COT | 0·51 | 1·78 | 0·79 | 0·65 | 0·09 |
| NIV vs COT | 0·51 | 0·24 | 0·45 | 0·84 | 0·42 |
| NIV vs HFNC | 0·42 | 3·33 | 0·57 | 0·85 | 0·01 |
| NIV+HFNC vs COT | 0·43 | 0·26 | 0·36 | 0·67 | 0·55 |
| NIV+HFNC vs HFNC | 0·51 | 0·18 | 0·46 | 0·90 | 0·34 |
| NIV+HFNC vs NIV | 0·35 | 2·49 | 0·80 | 0·57 | 0·01 |

Surface Under the Cumulative Ranking curve (SUCRA) and ranking probabilities for treatments

| Treatment | SUCRA | Probability of being the best (%) | Mean Rank |
| --- | --- | --- | --- |
| COT | 12·9 | 0·1 | 3·6 |
| HFNC | 27·9 | 0·2 | 3·1 |
| NIV | 78·3 | 46·4 | 1·7 |
| NIV+HFNC | 80·7 | 53·3 | 1·6 |

Supplemental Table 5: Network diagram, network estimates and absolute estimates evaluating the efficacy of the interventions for prevention of 7-day reintubation in critically ill adults in a sensitivity analysis on preventive support.

NIV: Noninvasive positive pressure ventilation, HFNC: High flow nasal oxygen, COT: Conventional oxygen therapy.


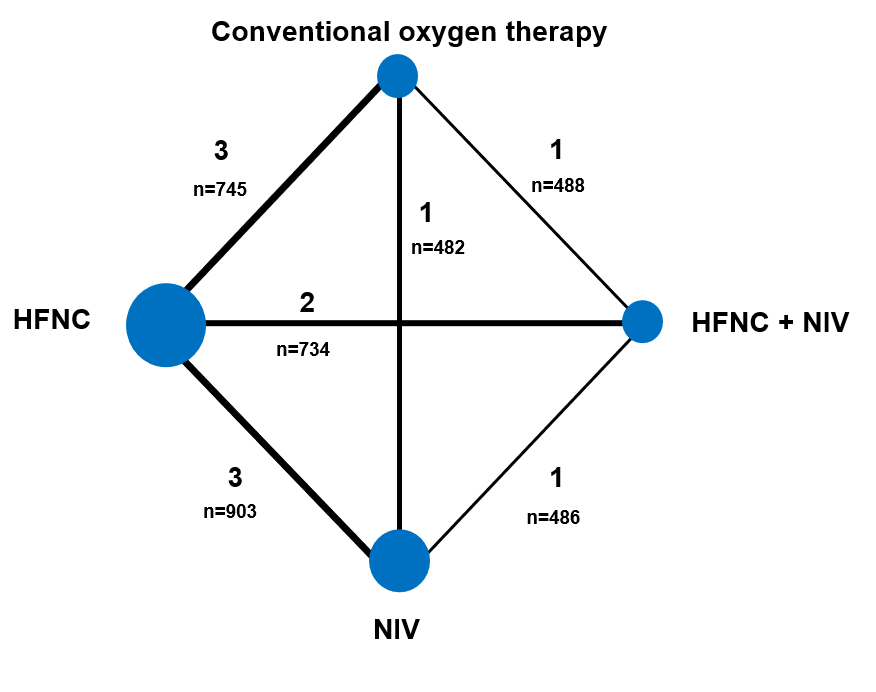


| **Comparison** | **Network Risk Ratio (95% CI)** | **p-value** | **Absolute risk difference (95% CI)** | **Number needed to treat** | **Grade** |
| --- | --- | --- | --- | --- | --- |
| NIV vs COT | 0·39 (0·14 ; 1·08) | 0·07 | -7·9  (-11·1 to 1·0) | NA | Low ^1,2^ |
| HFNC vs COT | 0·72 (0·28 ; 1·87) | 0·50 | -3·6 (-9·3 to 11·2) | NA | Low ^1,2^ |
| NIV vs HFNC | 0·54 (0·26 ; 1·10) | 0·09 | -6·2 (-9·9 to 1·3) | NA | Very low ^1,2,3^ |
| NIV + HFNC vs COT | 0·33 (0·12 ; 0·95) | 0·04 | -8·6 (-11·3 to -0·6) | 12 (9 to 156) | Moderate ^1^ |
| NIV + HFNC vs NIV | 0·86 (0·34 ; 2·18) | 0·94 | -1·8 (-8·4 to 15·1) | NA | Very low ^1,2,3^ |
| NIV + HFNC vs HFNC | 0·46 (0·21 ; 1·02) | 0·06 | -7·2 (-10·6 to 0·3) | NA | Low ^1,2^ |

^1^ Lowered one level for risk of bias

^2^ Lowered one level for imprecision as CIs don’t exclude harm.

^3^ Lowered for inconsistency

Supplemental Table 6: Direct and indirect estimates, tests of coherence, and Surface Under the Cumulative Ranking curve (SUCRA) for 7-day reintubation in critically ill adults in a sensitivity analysis on preventive support.

NIV: Noninvasive positive pressure ventilation, HFNC: High flow nasal oxygen, COT: Conventional oxygen therapy.

| **Comparison** | **Direct RR** | **Indirect RR** | **Network meta-analysis RR** | **Direct evidence proportion** | **P value for incoherence** |
| --- | --- | --- | --- | --- | --- |
| HFNC vs COT | 0·50 | 4·52 | 0·77 | 0·80 | 0·07 |
| NIV vs COT | 0·47 | 0·17 | 0·35 | 0·71 | 0·37 |
| NIV vs HFNC | 0·37 | 20·73 | 0·46 | 0·95 | 0·01 |
| NIV+HFNC vs COT | 0·43 | 0·13 | 0·34 | 0·78 | 0·39 |
| NIV+HFNC vs HFNC | 0·51 | 0·14 | 0·44 | 0·89 | 0·30 |
| NIV+HFNC vs NIV | 0·35 | 3·93 | 0·97 | 0·58 | 0·01 |

Surface Under the Cumulative Ranking curve (SUCRA) and ranking probabilities for treatments

| Treatment | SUCRA | Probability of being the best (%) | Mean Rank |
| --- | --- | --- | --- |
| COT | 22·9 | 4·4 | 3·3 |
| HFNC | 29·5 | 3·2 | 3·1 |
| NIV | 62·2 | 24·0 | 2·1 |
| NIV+HFNC | 85·3 | 68·4 | 1·4 |

Supplemental Table 7: Direct and indirect estimates, tests of coherence, and Surface Under the Cumulative Ranking curve (SUCRA) for 28-day mortality in critically ill adults.

NIV: Noninvasive positive pressure ventilation, HFNC: High flow nasal oxygen, COT: Conventional oxygen therapy.

| **Comparison** | **Direct RR** | **Indirect RR** | **Network meta-analysis RR** | **Direct evidence proportion** | **P value for incoherence** |
| --- | --- | --- | --- | --- | --- |
| HFNC vs COT | 0·55 | 11·8 | 1·32 | 0·72 | 0·02 |
| NIV vs COT | 0·60 | 0·06 | 0·41 | 0·83 | 0·14 |
| NIV vs HFNC | 0·21 | 14·1 | 0·31 | 0·91 | 0·01 |
| NIV+HFNC vs COT | 0·39 | 0·45 | 0·40 | 0·78 | 0·70 |
| NIV+HFNC vs HFNC | 0·40 | 0·03 | 0·30 | 0·89 | 0·16 |
| NIV+HFNC vs NIV | 0·31 | 7·8 | 0·97 | 0·64 | 0·01 |

Surface Under the Cumulative Ranking curve (SUCRA) and ranking probabilities for treatments

| Treatment | SUCRA | Probability of being the best (%) | Mean Rank |
| --- | --- | --- | --- |
| COT | 2·3 | 0·0 | 3·9 |
| HFNC | 46·5 | 7·2 | 2·6 |
| NIV | 85·6 | 65·1 | 1·4 |
| NIV+HFNC | 65·5 | 27·7 | 2·0 |

Supplemental Table 8: Network and absolute estimates evaluating the efficacy of the interventions for prevention of 28-day mortality in critically ill adults in a sensitivity analysis on preventive support.

NIV: Noninvasive positive pressure ventilation, HFNC: High flow nasal oxygen, COT: Conventional oxygen therapy.

**
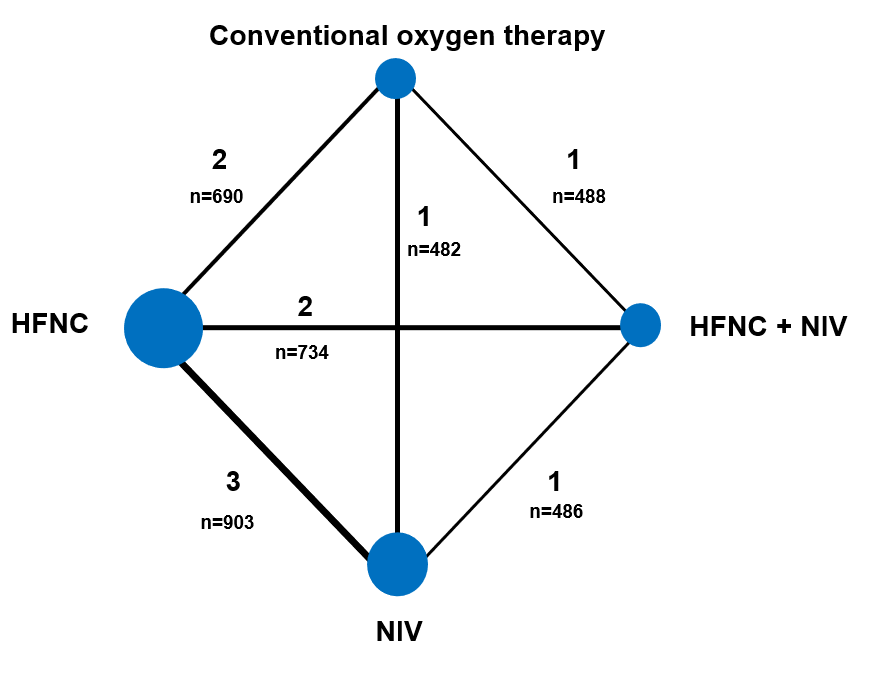
**

| **Comparison** | **Network Risk Ratio (95% CI)** | **p-value** | **Absolute risk difference (95% CI)** | **Number needed to treat** | **Grade** |
| --- | --- | --- | --- | --- | --- |
| NIV vs COT | 0·31 (0·08; 1·14) | 0·08 | -11·8 (-15·8 to 2·4) | NA | Low ^1,2^ |
| HFNC vs COT | 1·09 (0·31; 3·78) | 0·89 | 1·5 (-11·8 to 47·7) | NA | Very low ^1,2,3^ |
| NIV vs HFNC | 0·28 (0·11; 0·72) | <0·01 | -6·9 (-8·5 to -2·7) | 14 (12 to 37) | Moderate ^1,3,4^ |
| NIV + HFNC vs COT | 0·34 (0·08; 1·35) | 0·12 | -11·3 (-15·8 to 6·0) | NA | Low ^1,2^ |
| NIV + HFNC vs NIV | 1·09 (0·31; 3·86) | 0·89 | 0·8 (-5·9 to 24·6) | NA | Very low ^1,2,3^ |
| NIV + HFNC vs HFNC | 0·31 (0·10; 0·96) | 0·04 | -6·6 (-8·6 to -0·4) | 15 (12 to 262) | Moderate ^1^ |

^1^ Lowered one level for risk of bias

^2^ Lowered one level for imprecision as CIs don’t exclude harm.

^3^ Lowered for inconsistency

^4^ Increased one level for large effect.

Supplemental Table 9: Direct and indirect estimates, tests of coherence, and Surface Under the Cumulative Ranking curve (SUCRA) for 28-day mortality in critically ill adults in a sensitivity analysis on preventive support.

NIV: Noninvasive positive pressure ventilation, HFNC: High flow nasal oxygen, COT: Conventional oxygen therapy.

| **Comparison** | **Direct RR** | **Indirect RR** | **Network meta-analysis RR** | **Direct evidence proportion** | **P value for incoherence** |
| --- | --- | --- | --- | --- | --- |
| HFNC vs COT | 0·56 | 17·86 | 1·09 | 0·81 | 0·03 |
| NIV vs COT | 0·48 | 0·06 | 0·31 | 0·78 | 0·21 |
| NIV vs HFNC | 0·21 | 37·22 | 0·28 | 0·94 | 0·01 |
| NIV+HFNC vs COT | 0·45 | 0·08 | 0·34 | 0·83 | 0·36 |
| NIV+HFNC vs HFNC | 0·40 | 0·04 | 0·31 | 0·89 | 0·22 |
| NIV+HFNC vs NIV | 0·31 | 13·42 | 1·09 | 0·66 | 0·01 |

Surface Under the Cumulative Ranking curve (SUCRA) and ranking probabilities for treatments

| Treatment | SUCRA | Probability of being the best (%) | Mean Rank |
| --- | --- | --- | --- |
| COT | 4·2 | 0·3 | 3·9 |
| HFNC | 46·4 | 8·8 | 2·7 |
| NIV | 81·5 | 57·2 | 1·6 |
| NIV+HFNC | 68·0 | 33·7 | 1·9 |

Supplemental Table 10: Network diagram, network estimates and absolute estimates evaluating the efficacy of the interventions for ICU length of stay in critically ill adults.

NIV: Noninvasive positive pressure ventilation, HFNC: High flow nasal oxygen, COT: Conventional oxygen therapy.


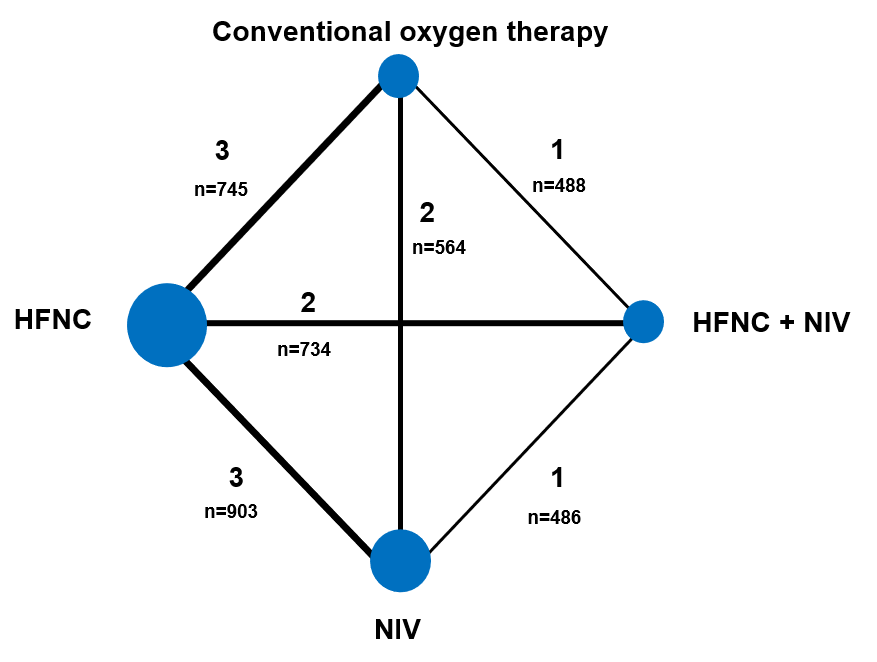


| **Comparison** | **Network Mean Difference (days, 95% CI)** | **p-value** | **Grade** |
| --- | --- | --- | --- |
| NIV vs COT | -0·05 (-0·39 ; 0·30) | 0·77 | Very low ^1,2,3^ |
| HFNC vs COT | -0·12 (-0·40 ; 0·17) | 0·41 | Very low ^1,2,3^ |
| NIV vs HFNC | 0·07 (-0·20 ; 0·34) | 0·61 | Very low ^1,2,3^ |
| NIV + HFNC vs COT | -0·09 (-0·46 ; 0·28) | 0·62 | Very low ^1,2,3^ |
| NIV + HFNC vs NIV | -0·02 (-0·45 ; 0·41) | 0·92 | Very low ^1,2,3^ |
| NIV + HFNC vs HFNC | -0·04 (-0·41 ; 0·32) | 0·83 | Very low ^1,2,3^ |

^1^ Lowered one level for risk of bias

^2^ Lowered one level for imprecision as CIs don’t exclude harm.

^3^ Lowered for heterogeneity

Supplemental Table 11: Direct and indirect estimates, tests of coherence, and SUCRA table evaluating the efficacy of the interventions for ICU length of stay.

NIV: Noninvasive positive pressure ventilation, HFNC: High flow nasal oxygen, COT: Conventional oxygen therapy.

| **Comparison** | **Direct MD (days)** | **Indirect MD (days)** | **Network meta-analysis MD (days)** | **Direct evidence proportion** | **P value for incoherence** |
| --- | --- | --- | --- | --- | --- |
| HFNC vs COT | -0·12 | -0·11 | -0·12 | 0·88 | 0·97 |
| NIV vs COT | -0·05 | -0·06 | -0·05 | 0·59 | 0·98 |
| NIV vs HFNC | 0·06 | 0·15 | 0·07 | 0·90 | 0·85 |
| NIV+HFNC vs COT | -0·07 | -0·13 | -0·09 | 0·88 | 0·87 |
| NIV+HFNC vs HFNC | -0·01 | -0·15 | -0·04 | 0·77 | 0·77 |
| NIV+HFNC vs NIV | -0·04 | 0·00 | -0·11 | 0·62 | 0·78 |

Surface Under the Cumulative Ranking curve (SUCRA) and ranking probabilities for treatments

| Treatment | SUCRA | Probability of being the best (%) | Mean Rank |
| --- | --- | --- | --- |
| COT | 34·3 | 3·3 | 3·0 |
| HFNC | 27·0 | 1·1 | 2·8 |
| NIV | 88·9 | 73·4 | 1·5 |
| NIV+HFNC | 49·8 | 22·2 | 2·5 |

Supplemental Table 12: Network diagram, network estimates and absolute estimates evaluating the efficacy of the interventions for ICU length of stay in critically ill adults in a sensitivity analysis on preventive support.

NIV: Noninvasive positive pressure ventilation, HFNC: High flow nasal oxygen, COT: Conventional oxygen therapy.


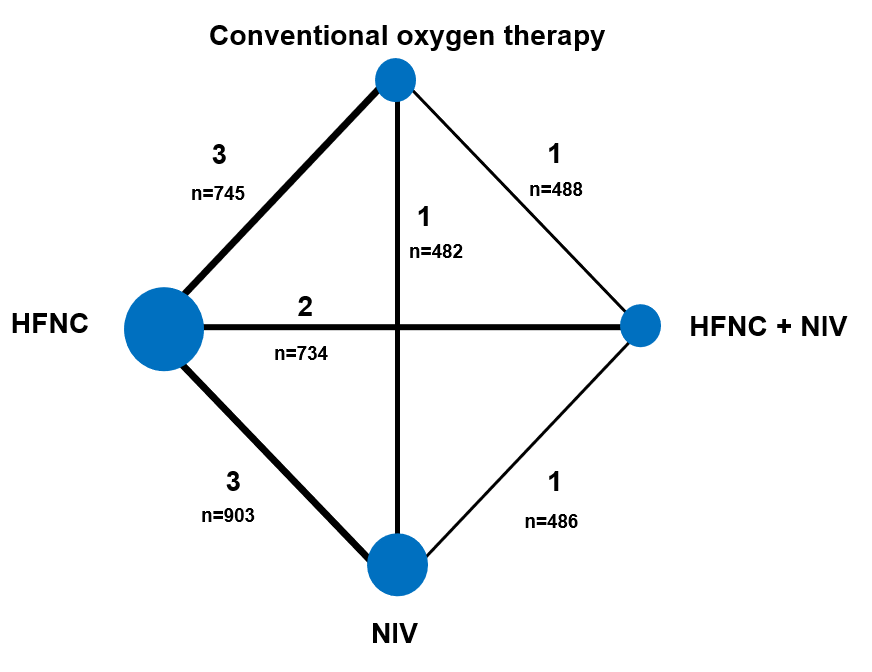


| Comparison | Network Mean Difference (days, 95% CI) | p-value | Grade |
| --- | --- | --- | --- |
| NIV vs COT | -0·02 (-0·39 ; 0·35) | 0·91 | Very low ^1,2,3^ |
| HFNC vs COT | -0·06 (-0·40 ; 0·28) | 0·72 | Very low ^1,2,3^ |
| NIV vs HFNC | 0·04 (-0·21 ; 0·29) | 0·75 | Very low ^1,2,3^ |
| NIV + HFNC vs COT | -0·04 (-0·50 ; 0·41) | 0·85 | Very low ^1,2,3^ |
| NIV + HFNC vs NIV | -0·02 (-0·45 ; 0·41) | 0·92 | Very low ^1,2,3^ |
| NIV + HFNC vs HFNC | 0·02 (-0·41 ; 0·44) | 0·94 | Very low ^1,2,3^ |

^1^ Lowered one level for risk of bias

^2^ Lowered one level for imprecision as CIs don’t exclude harm.

^3^ Lowered for inconsistency

Supplemental Table 13: Direct and indirect estimates, tests of coherence, and SUCRA table evaluating the efficacy of the interventions for ICU length of stay in a sensitivity analysis on preventive support.

NIV: Noninvasive positive pressure ventilation, HFNC: High flow nasal oxygen, COT: Conventional oxygen therapy.

| **Comparison** | **Direct MD (days)** | **Indirect MD (days)** | **Network meta-analysis MD (days)** | **Direct evidence proportion** | **P value for incoherence** |
| --- | --- | --- | --- | --- | --- |
| HFNC vs COT | -0·04 | -0·34 | -0·08 | 0·88 | 0·60 |
| NIV vs COT | -0·07 | 0·20 | -0·04 | 0·59 | 0·59 |
| NIV vs HFNC | 0·10 | -0·13 | 0·09 | 0·97 | 0·74 |
| NIV+HFNC vs COT | -0·07 | 0·20 | -0·04 | 0·88 | 0·71 |
| NIV+HFNC vs HFNC | 0·02 | 0·11 | 0·04 | 0·77 | 0·86 |
| NIV+HFNC vs NIV | 0·00 | -0·28 | -0·06 | 0·80 | 0·63 |

Surface Under the Cumulative Ranking curve (SUCRA) and ranking probabilities for treatments

| Treatment | SUCRA | Probability of being the best (%) | Mean Rank |
| --- | --- | --- | --- |
| COT | 44·9 | 15·5 | 2·6 |
| HFNC | 43·0 | 10·0 | 3·0 |
| NIV | 62·5 | 38·3 | 2·1 |
| NIV+HFNC | 49·6 | 36·2 | 2·5 |

Supplemental Table 14: Network diagram and network estimates evaluating the efficacy of the interventions for the Hospital length of stay in critically ill adults.

NIV: Noninvasive positive pressure ventilation, HFNC: High flow nasal oxygen, COT: Conventional oxygen therapy.


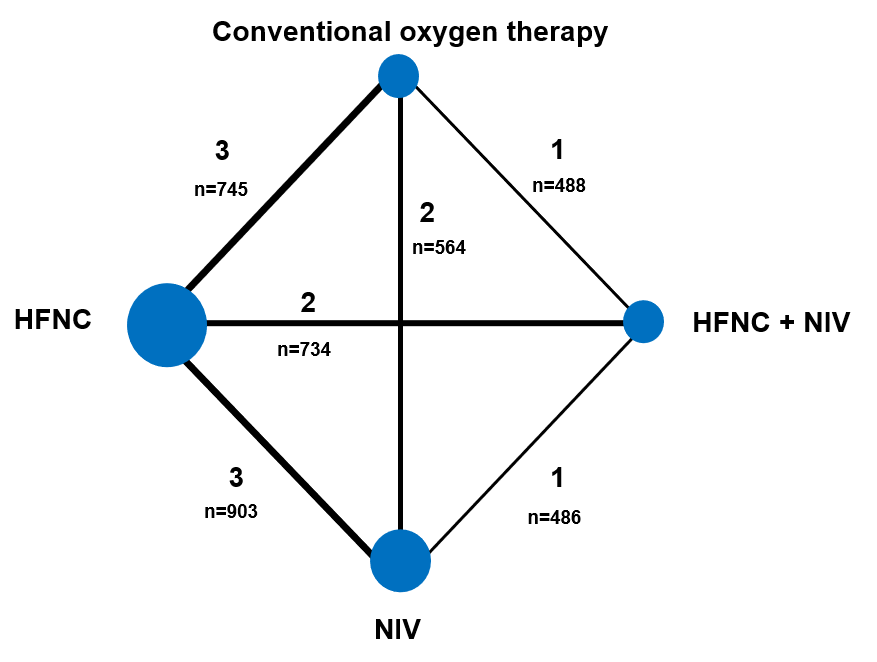


| **Comparison** | **Network MD (days, 95% CI)** | **p-value** | **Grade** |
| --- | --- | --- | --- |
| NIV vs COT | 0·02 (-0·17 ; 0·21) | 0·84 | Low ^1,2^ |
| HFNC vs COT | -0·08 (-0·26 ; 0·10) | 0·39 | Low ^1,2^ |
| NIV vs HFNC | 0·10 (-0·05 ; 0·24) | 0·19 | Low ^1,2^ |
| NIV + HFNC vs COT | 0·00 (-0·18 ; 0·19) | 0·97 | Low ^1,2^ |
| NIV + HFNC vs NIV | -0·02 (-0·20 ; 0·16) | 0·86 | Low ^1,2^ |
| NIV + HFNC vs HFNC | 0·08 (-0·07 ; 0·24) | 0·29 | Low ^1,2^ |

^1^ Lowered one level for risk of bias

^2^ Lowered one level for imprecision as CIs don’t exclude harm.

Supplemental Table 15: Direct and indirect estimates, tests of coherence, and SUCRA table evaluating the efficacy of the interventions for Hospital length of stay.

NIV: Noninvasive positive pressure ventilation, HFNC: High flow nasal oxygen, COT: Conventional oxygen therapy.

| **Comparison** | **Direct MD (days)** | **Indirect MD (days)** | **Network meta-analysis MD (days)** | **Direct evidence proportion** | **P value for incoherence** |
| --- | --- | --- | --- | --- | --- |
| HFNC vs COT | 0·04 | -0·31 | -0·03 | 0·81 | 0·33 |
| NIV vs COT | 0·04 | 0·40 | 0·10 | 0·85 | 0·35 |
| NIV vs HFNC | 0·12 | NA | 0·12 | 1·00 | NA |
| NIV + HFNC vs COT | 0·09 | NA | 0·09 | 1·00 | NA |
| NIV + HFNC vs NIV | 0·05 | 0·41 | 0·11 | 0·82 | 0·31 |
| NIV + HFNC vs HFNC | 0·06 | -0·32 | -0·01 | 0·82 | 0·29 |

Surface Under the Cumulative Ranking curve (SUCRA) and ranking probabilities for treatments

| Treatment | SUCRA | Probability of being the best (%) | Mean Rank |
| --- | --- | --- | --- |
| COT | 45·3 | 23·6 | 2·6 |
| HFNC | 77·7 | 53·1 | 1·7 |
| NIV | 43·0 | 10·1 | 2·7 |
| NIV+HFNC | 33·9 | 13·2 | 2·7 |

Supplemental Table 16: Network diagram and network estimates evaluating the efficacy of the interventions for the Hospital length of stay in critically ill adults in a sensitivity analysis on preventive support.

NIV: Noninvasive positive pressure ventilation, HFNC: High flow nasal oxygen, COT: Conventional oxygen therapy.


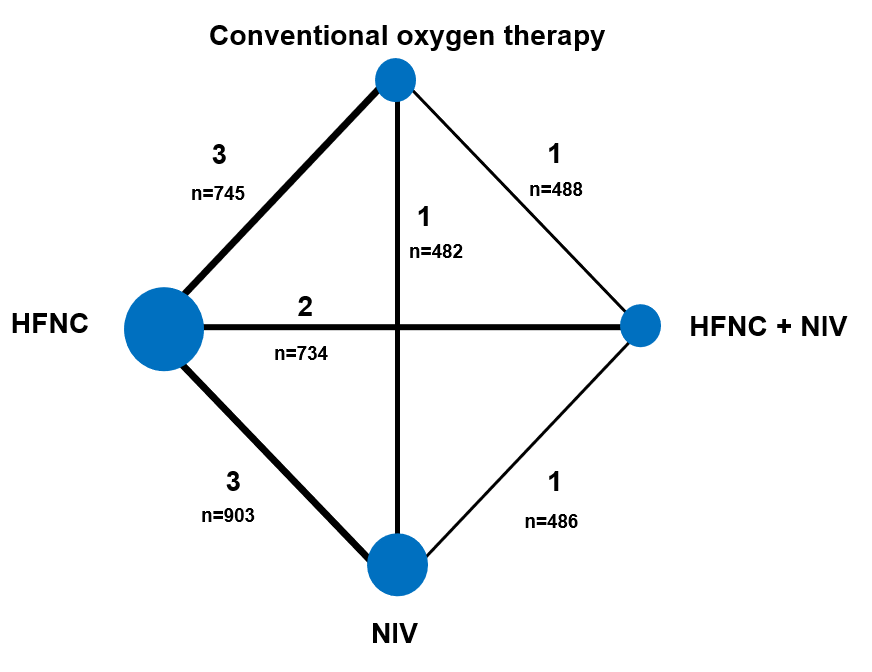


| **Comparison** | **Network Mean Difference (days, 95% CI)** | **p-value** | **Grade** |
| --- | --- | --- | --- |
| NIV vs COT | 0·06 (-0·09 ; 0·21) | 0·45 | Low ^1,2^ |
| HFNC vs COT | 0·01 (-0·15 ; 0·17) | 0·94 | Low ^1,2^ |
| NIV vs HFNC | 0·05 (-0·05 ; 0·15) | 0·32 | Low ^1,2^ |
| NIV + HFNC vs COT | 0·09 (-0·07 ; 0·24) | 0·29 | Low ^1,2^ |
| NIV + HFNC vs NIV | 0·03 (-0·12 ; 0·17) | 0·71 | Low ^1,2^ |
| NIV + HFNC vs HFNC | 0·08 (-0·07 ; 0·22) | 0·29 | Low ^1,2^ |

^1^ Lowered one level for risk of bias

^2^ Lowered one level for imprecision as CIs don’t exclude harm.

Supplemental Table 17: Direct and indirect estimates, tests of coherence, and SUCRA table evaluating the efficacy of the interventions for Hospital length of stay.

NIV: Noninvasive positive pressure ventilation, HFNC: High flow nasal oxygen, COT: Conventional oxygen therapy.

| **Comparison** | **Direct MD (days)** | **Indirect MD (days)** | **Network meta-analysis MD (days)** | **Direct evidence proportion** | **P value for incoherence** |
| --- | --- | --- | --- | --- | --- |
| HFNC vs COT | 0·04 | -0·31 | -0·03 | 0·81 | 0·33 |
| NIV vs COT | 0·04 | 0·40 | 0·10 | 0·85 | 0·35 |
| NIV vs HFNC | 0·12 | NA | 0·12 | 1·00 | NA |
| NIV+HFNC vs COT | 0·09 | NA | 0·09 | 1·00 | NA |
| NIV+HFNC vs HFNC | 0·05 | 0·41 | 0·11 | 0·82 | 0·31 |
| NIV+HFNC vs NIV | 0·06 | -0·32 | -0·01 | 0·82 | 0·29 |

Surface Under the Cumulative Ranking curve (SUCRA) and ranking probabilities for treatments

| Treatment | SUCRA | Probability of being the best (%) | Mean Rank |
| --- | --- | --- | --- |
| COT | 45·3 | 23·6 | 2·6 |
| HFNC | 77·7 | 53·1 | 1·7 |
| NIV | 43·0 | 10·1 | 2·7 |
| NIV+HFNC | 33·9 | 13·2 | 2·7 |

Supplemental Table 18: Network diagram and network estimates evaluating the efficacy of the interventions for the Radiological Atelectasis Score (RAS) in critically ill. adults.

HFNO: High flow nasal oxygen, COT: Conventional oxygen therapy.


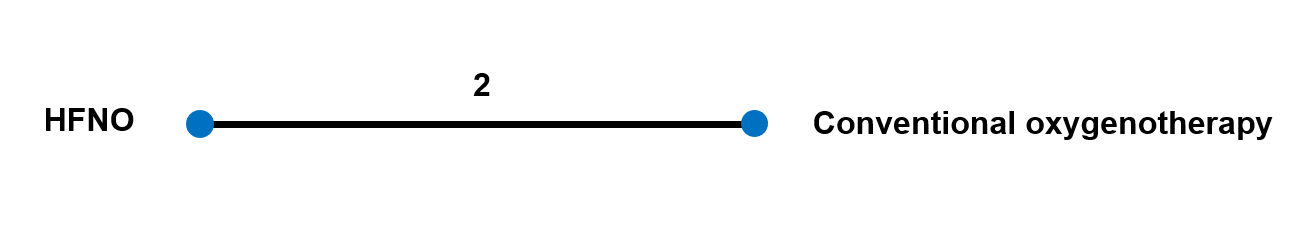


| **Comparison** | **Mean Difference (RAS score, 95% CI)** | **p-value** | **Grade** |
| --- | --- | --- | --- |
| HFNO vs COT | -0·40 (-1·20 ; 0·41) | 0·33 | Low ^1,2^ |

^1^ Lowered one level for risk of bias

^2^ Lowered one level for imprecision as CIs don’t exclude harm.

Supplemental Table 19: Direct and indirect estimates, tests of coherence, and SUCRA table evaluating the efficacy of the interventions for Radiological Atelectasis Score (RAS).

HFNO: High flow nasal oxygen, COT: Conventional oxygen therapy.

| **Comparison** | **Direct MD (RAS)** | **Indirect MD (RAS)** | **Network meta-analysis MD (RAS)** | **Direct evidence proportion** | **P value for incoherence** |
| --- | --- | --- | --- | --- | --- |
| HFNO vs COT | -0·40 | NA | -0·40 | 1·00 | NA |

Surface Under the Cumulative Ranking curve (SUCRA) and ranking probabilities for treatments

| Treatment | SUCRA | Probability of being the best (%) | Mean Rank |
| --- | --- | --- | --- |
| COT | 12·5 | 12·5 | 1·9 |
| HFNO | 87·5 | 87·5 | 1·1 |

**Results obtained from queries to the authors**

Four queries were sent to the authors, and all of them were answered:

- Thille 2022: In the original publication, the results on all outcomes were presented for both patients with overweight and obesity. The results on the obesity subgroup were asked to Pr Frat.
- Hernandez 2024: The record was identified through ClinicalTrials.gov. Since the RCT was not yet published when the study started, the results were asked to Pr Hernandez. Pr Hernandez sent an early version of the submitted manuscript.
- De Jong 2023 and Jaber 2024: The results on all outcomes were double checked with the Medical Information Department of our University Hospital.
